# Pain in adults with cerebral palsy: A systematic review

**DOI:** 10.1111/dmcn.16254

**Published:** 2025-02-12

**Authors:** Jennifer M. Ryan, Jessica Burke, Rachel Byrne, Emily Capellari, Adrienne Harvey, Neil E. O'Connell, Donna Omichinski, Elisabet Rodby‐Bousquet, Mark Peterson, The Adult CP Clinical Practice Guideline Working Group, Jan Willem Gorter, Ashley Harris Whaley, Christine Imms, Christina M Marciniak, Karen Brady

**Affiliations:** ^1^ CP‐Life Research Centre, School of Physiotherapy Royal College of Surgeons in Ireland Dublin Ireland; ^2^ Cerebral Palsy Foundation New York USA; ^3^ Department of Physical Medicine and Rehabilitation Medicine University of Michigan Ann Arbor USA; ^4^ Neurodisability and Rehabilitation Murdoch Children's Research Institute Melbourne Australia; ^5^ Department of Health Sciences, Centre for Wellbeing Across the Lifecourse Brunel University London London UK; ^6^ Department of Clinical Sciences Lund, Ortopaedics Lund University Lund Sweden; ^7^ Centre for Clinical Research Uppsala University ‐ Region Västmanland Västerås Sweden

## Abstract

**Aim:**

To describe the prevalence and incidence of pain, identify prognostic factors for pain, determine psychometric properties of tools to assess pain, and evaluate effectiveness of interventions for reducing pain among adults with cerebral palsy (CP).

**Method:**

Six databases were searched to identify studies published since 1990 in any language that met eligibility criteria defined for each objective. Titles, abstracts, and full texts were screened by two independent reviewers.

**Results:**

Sixty‐three studies were identified; 47 reporting prevalence, 28 reporting prognostic factors, four reporting psychometric properties, five evaluating intervention effectiveness. Pain prevalence ranged from 24% to 89%. Prevalence was higher among adults with CP than in adults without it. Communication function, sex, and age were prognostic factors for pain prevalence. Numerical, verbal, and pictorial rating scales were valid for assessing pain intensity in adults with CP. Pharmacological and surgical interventions had no effect on pain. An active lifestyle and sports intervention reduced pain in adults with CP compared with usual care.

**Interpretation:**

Many adults with CP experience pain, although prevalence estimates vary considerably. The quality of evidence for prognostic factors and interventions is very low to low. There is a lack of evidence about effective pain management among adults with CP.

**Abbreviations**
CFCS: Communication Function Classification System
RCT: randomized controlled trial
SF‐36: 36‐Item Short Form Survey

**What this paper adds**

- The prevalence of chronic pain, defined as pain for longer than 3 months, was 75% to 78%.
- There was low certainty in the evidence that pain is more prevalent in adults with cerebral palsy.
- There was moderate certainty that pain is more prevalent in adults with better communication.
- There was low certainty that the prevalence of pain does not differ across Gross Motor Function Classification System levels.
- There was very low to low certainty for the effectiveness of pharmacological and non‐pharmacological interventions to reduce pain.

Pain is one of the most commonly reported comorbidities by children and adults with cerebral palsy (CP).^1^, ^2^ Pain is defined as ‘an unpleasant sensory and emotional experience associated with, or resembling that associated with, actual or potential tissue damage’.^3^ The experience of pain may have wide‐ranging consequences for a person with CP. Pain is a strong predictor of reduced quality of life among children and adults with CP,^2^, ^4^, ^5^, ^6^ and pain in childhood is associated with reduced quality of life in adolescence.^5^ Pain also negatively affects sleep, behaviour, and activity among children and adults with CP.^7^, ^8^, ^9^, ^10^, ^11^ Further, adults with CP who experience pain have poorer psychological and employment outcomes.^6^, ^12^, ^13^

Given the impact of pain on the lives of adults with CP, it is important to understand the burden of pain and factors that influence the development of pain to inform treatment recommendations and management of patients. A 2019 systematic review identified that between 14% and 76% of children and young adults with CP report pain.^2^ Prognostic factors for pain prevalence and intensity in children and young adults with CP included age, female sex, severe motor impairment, reduced mobility, CP subtype, and musculoskeletal complications.^2^, ^11^, ^14^, ^15^ A meta‐analysis of 16 studies published up to 2018 reported the prevalence of any pain in adults with CP was 65%.^1^ In 2021, a meta‐analysis of individual participant data from 14 studies published between 2000 and 2016 estimated the prevalence of pain in adults with CP was 70%.^16^ Pain prevalence was higher in females than males and in adults with CP classified in Gross Motor Function Classification System (GMFCS) levels II and IV compared with level I. Studies describing pain prevalence or incidence that were published after 2018 have not been synthesized, resulting in an incomplete understanding of the current evidence on pain in adults with CP. Further, while a systematic review examined the evidence for pharmacological, surgical, and rehabilitative interventions to manage pain in children with CP,^17^ no review has summarized evidence of the effectiveness of such interventions to reduce pain in adults with CP.

Therefore, the aim of this study was to synthesize evidence relating to pain in adults with CP. All types of pain (e.g. nociceptive, neuropathic) and pain duration (e.g. chronic, acute) were of interest.

The objectives were to (1) describe the prevalence and incidence of pain among adults with CP and compare them with adults without CP; (2) identify prognostic factors for pain presence and pain intensity in adults with CP; (3) determine the psychometric properties and feasibility of the tools used to assess pain among adults with CP; and (4) evaluate the safety and effectiveness of interventions for reducing pain in adults with CP, including non‐pharmacological and pharmacological interventions.

## METHOD

The protocol for this review was registered on Open Science Framework (https://doi.org/10.17605/OSF.IO/RMXUF). The methods were guided by the JBI Manual for Evidence Synthesis.^18^ Results are reported using the Preferred Reporting Items for Systematic Reviews and Meta‐analysis (PRISMA) and Meta‐Analyses of Observational Studies in Epidemiology (MOOSE) statements.

### Literature search

An experienced information specialist developed and conducted comprehensive searches using the online databases PubMed, Embase, Cochrane CENTRAL, Cochrane Database of Systematic Reviews, CINAHL, and PsycINFO. A single search was conducted in each database to identify studies for all objectives. Reference lists of relevant systematic reviews and included studies were searched for additional articles. An example search strategy for PubMed is in Appendix S1.

### Eligibility criteria

Eligibility criteria for each objective are outlined in Appendix S2 according to the following frameworks as applicable to each question: CoCoPop (Condition, Context, Population), PEO (Population, Exposure, Outcome), PICO (Population, Instrument, Construct, Outcome), PICO (Population, Intervention, Comparator, Outcome). Studies describing all types of pain (e.g. nociceptive, neuropathic) and describing acute or chronic pain were included. Studies published since 1990 in any language were included. Conference abstracts, guidelines, editorials, commentaries and opinion pieces, protocols, narrative reviews, case studies, case reports, and other study designs reporting data on fewer than five individuals with CP were excluded. Systematic reviews that directly addressed our question of interest and were conducted in the previous 3 years were eligible for inclusion; however, we did not identify any systematic reviews meeting these criteria.

#### Population

For all objectives, the population was defined as adults with CP aged 16 years or older. However, where studies included people aged 16 years and 17 years, they were included only if they also included adults aged 18 years and older. Where studies included mixed populations (i.e. both children and adults or adults with CP and other conditions), they were included if data on adults with CP could be extracted.

#### Additional criteria by objective

##### Objective 1

The condition was prevalence or incidence of pain. The context was any country worldwide and any setting (e.g. population/community‐based or hospital‐based). Cohort and cross‐sectional studies were included. All intervention study designs were excluded (e.g. randomized controlled trials [RCTs], quasi‐experimental).

##### Objective 2

Exposures were any modifiable or non‐modifiable socio‐demographic or clinical factor, whose association with pain was examined, such as sex, GMFCS level, musculoskeletal complications. Factors assessed at any time during childhood or adulthood were included. The outcome was prevalent or incident pain or pain intensity. Cohort, case–control, and cross‐sectional studies were included. All intervention study designs were excluded (e.g. RCT, quasi‐experimental).

##### Objective 3

All studies describing the validity, reliability, responsiveness, or feasibility of patient‐ or clinician‐reported instruments that assessed pain presence, pain intensity, pain location, or pain interference were included. Any quantitative study design was included. Studies that only used the measurement instrument as an outcome measure were excluded from this objective. Studies that duplicated validation data of an instrument in a previous study (i.e. did not present new measurement property data), and studies that aimed to translate and validate an instrument in a language other than English, were also excluded as they did not provide new data about the psychometric properties of the instrument.

##### Objective 4

Studies examining the effect of any intervention that aimed to effect prognostic factors for pain or pain intensity were included. Interventions could include, but were not limited to, pharmacological interventions, surgical interventions, physical or psychological interventions. Eligible comparators included usual care, no intervention, a modified version of the intervention, or a different intervention. Outcomes were pain presence, pain intensity, pain duration or frequency, and adverse events. Studies that recorded pain as an adverse event only, rather than being assessed before and after the intervention using a standardized method, were excluded because they did not provide sufficient data to determine the effect of the intervention on pain. RCTs, controlled before‐ and after‐studies, uncontrolled before‐ and after‐studies, and interrupted time series were included.

### Study selection process

Two independent reviewers screened titles and abstracts using a screening checklist with a third reviewer resolving discrepancies between the reviewers. Two reviewers then independently screened full texts using a separate screening checklist for each review question that was piloted before use. A third reviewer resolved discrepancies between the reviewers. We used a machine translation engine (Google Translate) to translate non‐English language papers into English.

### Data extraction

A single reviewer extracted items for included studies using a standardized data extraction template that was piloted before use. A second reviewer verified data. For all studies, data on study characteristics (i.e. study design, country or countries, year[s] of data collection), source population, eligibility criteria, study setting, and the following characteristics of participants were extracted: age, sex, CP subtype, GMFCS level, Manual Ability Classification System level, Communication Function Classification System (CFCS) level, Eating and Drinking Ability Classification System level, intellectual disability, socioeconomic status, ethnicity, body mass index. Where classification systems were not used to assess function, related data describing function such as ambulatory status or tube‐feeding status were extracted.

Data describing the prevalence and incidence of pain for adults with CP, using raw data for denominators and numerators where available, were extracted. Data on type of pain, location of pain, duration of pain, pain intensity, pain frequency, and how it was assessed were extracted. Type of pain was categorized as nociceptive, nociplastic, neuropathic (definable nerve injury), and mixed. If available, data on the prevalence or incidence of pain in adults without CP or the general population, and ratios comparing the incidence or prevalence of pain between adults with CP and adult without CP or the general population, with associated confidence intervals and *p*‐values, were extracted. Data about each prognostic factor, how it was assessed, and follow‐up or study duration were extracted. Data on associations between prognostic factors and outcomes, such as risk ratio, odds ratio, risk difference, correlation coefficients, with associated confidence intervals and *p*‐values, were extracted. Adjusted estimates were extracted. Unadjusted estimates were extracted only when no other data were available.

When examining psychometric properties, the following data were extracted: data on the instrument(s) including specific information about subscales if only parts of a larger instrument were used, construct assessed, mode of administration, validity, reliability, responsiveness, and feasibility were extracted. Associations were categorized as poor (*r* < 0.30), moderate (*r* = 0.30–0.49), good (*r* = 0.50–0.69), and excellent (*r* ≥ 0.70).

When examining effectiveness, data on the interventions, comparators, outcomes, adverse events, time‐points for assessments, and effect estimates with 95% confidence intervals (CI) and *p*‐values (e.g. mean difference or odds ratios) were extracted. If confidence intervals were not reported, exact *p*‐values were described. Short‐term effect was defined as 0 to 3 months post‐intervention, medium‐term as 3 to 6 months post‐intervention, and long‐term as more than 6 months post‐intervention.

### Quality appraisal

Quality appraisal was conducted independently by two reviewers using a JBI critical appraisal checklist or the COSMIN risk of bias checklist; conflicts were resolved through discussion. Specifically, the following JBI critical appraisal checklists were used for each study design: prevalence studies checklist for studies reporting prevalence or incidence; cohort studies checklist for cohort studies examining the association between prognostic factors and pain; cross‐sectional studies checklist for cross‐sectional studies reporting associations between prognostic factors and pain and for studies comparing prevalence of pain between adults with and without CP; RCT checklist for RCTs examining the effect of an intervention; quasi‐experimental studies checklist for quasi‐experimental studies examining the effect of an intervention. The COSMIN risk of bias checklist was used to appraise studies reporting validity of instruments to assess pain. Studies were assigned an overall rating of high, low, or unclear risk of bias by taking the lowest rating of any question (i.e. ‘the worst score counts’ principle). For example, for the COSMIN checklist, if one item was rated as ‘inadequate’, the overall methodological quality of that study was rated as having high risk of bias.

### Certainty in the findings

The Grading of Recommendations Assessment, Development and Evaluation (GRADE) approach was used to assess the certainty of evidence (see Appendix S3 for criteria used in assessment). Comparisons of interest were as follows: (1) adults with CP compared with adults without CP (outcome: pain presence); (2) adults across GMFCS levels (outcome: pain presence); (3) adults across CFCS levels (outcome: pain presence); (4) an index test compared with a reference method (outcome: pain presence or pain intensity); (5) pharmacological intervention compared with placebo, no intervention, or usual care (outcome: pain presence or pain intensity); (6) non‐pharmacological intervention compared with placebo, no intervention, or usual care (outcome: pain presence or pain intensity); (7) surgical intervention compared with placebo, no intervention, or usual care (outcome: pain presence or pain intensity).

### Synthesis

A descriptive synthesis of the evidence was conducted. Summaries of the volume of information gleaned and included studies are presented in the tables and text. Included studies are categorized according to the research question. Detailed summary of findings tables with study results including effect estimates, 95% CI, and *p*‐values if reported are provided. GRADE tables are provided with a summary of results of each comparison and confidence in the evidence.

## RESULTS

Study selection is described in Figure S1. Searches of PubMed, Embase, Cochrane CENTRAL, Cochrane Database of Systematic Reviews, CINAHL, and PsycINFO up to 29 April 2024 identified 4652 records. Twenty‐five additional references were identified from manual searching of reference lists of systematic reviews or reference lists of included studies. After removal of duplicates, there were 1881 records. Of these, 1397 were excluded after title and abstract screening, six full texts could not be retrieved, and 478 full texts were obtained. A further 415 records were excluded after full‐text screening resulting in 63 reports. Characteristics of included studies are described in Table S1.

Appraisals of study quality are provided in Tables S2–S8. Of the studies reporting prevalence, 46 were at high risk of bias and one had unclear risk of bias.^10^ All studies comparing prevalence between adults with and without CP were at high risk of bias. All studies reporting prognostic factors for pain were at high risk of bias. Three studies describing psychometric properties of tools were at high risk of bias and one had unclear risk of bias.^19^ All studies evaluating effectiveness of interventions were at high risk of bias.

### Epidemiology and characteristics of pain

Forty‐seven cross‐sectional studies, including 29 814 adults with CP, described the prevalence of pain. No studies reported incidence. Sample size ranged from 17 to 8796. Of these, 14 (30%) reported data on adults with CP living in the USA. Mean age when reported ranged from 21 years 2 months to 54 years 6 months. One study only included adults older than 65 years. The percentage of females in each sample ranged from 18% to 100% (median 47%). Twenty‐one studies (45%) included adults with CP classified in all GMFCS levels. Five (11%) included adults with CP in GMFCS levels I to III only and two included adults in GMFCS levels IV/V or wheelchair users only.

### Prevalence of pain

**FIGURE 1:**
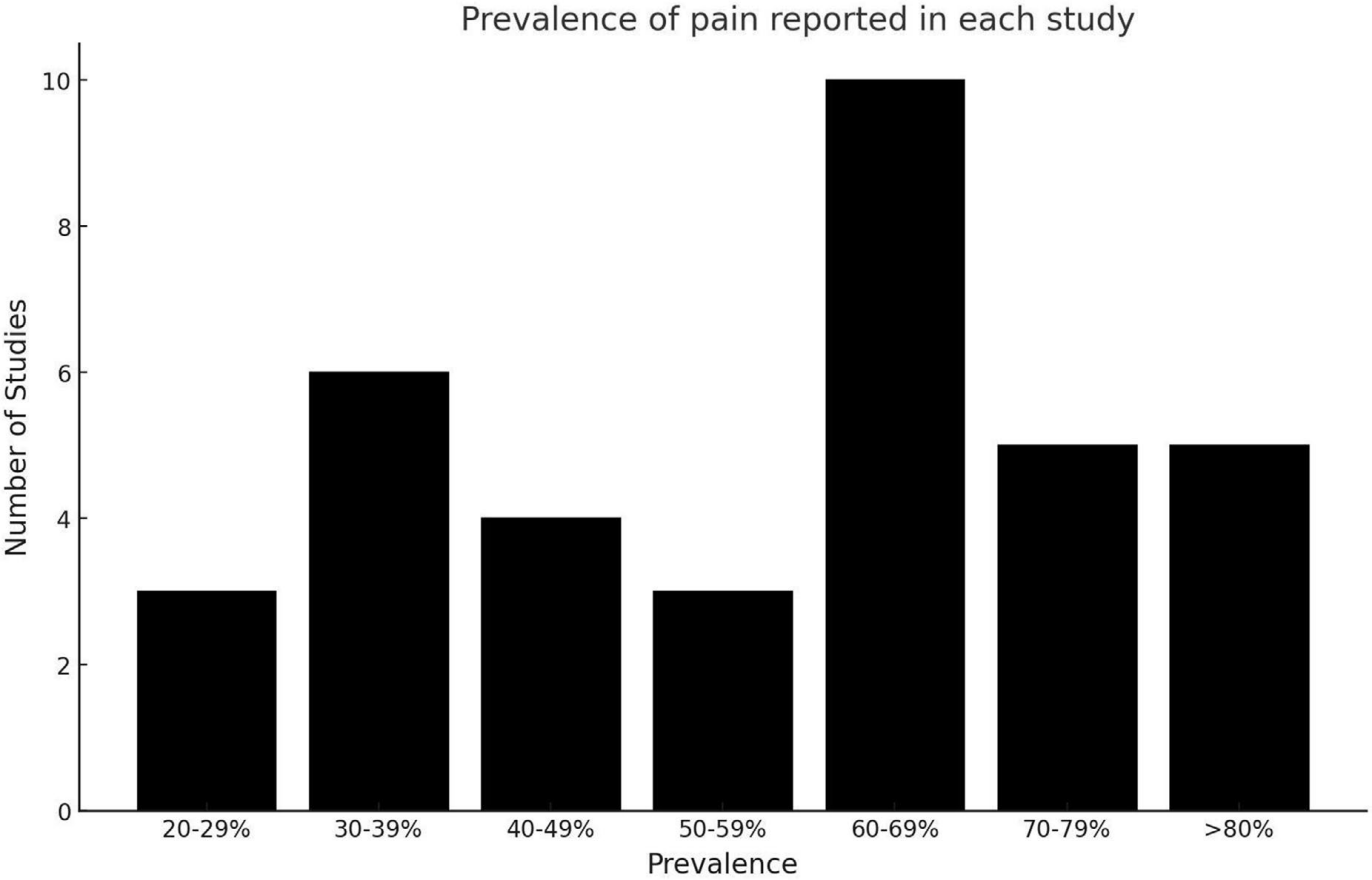
Prevalence of pain reported in each study.

Evidence for prevalence of pain, pain location, duration, and frequency among adults with CP is presented in Table S9 and Table 1. Prevalence of pain ranged from 24%^20^, ^21^ to 89%.^22^ Figure 1 describes the distribution of prevalence across studies. Prevalence was similar between clinic‐ and population‐based settings and did not change when studies of adults with CP who had received orthopaedic surgery or selective dorsal rhizotomy in childhood were excluded.^20^, ^23^, ^24^, ^25^, ^26^, ^27^, ^28^ Thirty‐one per cent of studies stated that pain was both self‐ and proxy‐reported, 29% stated that pain was self‐reported only, and 3% stated that pain was proxy‐reported only. The remaining 37% of studies did not clearly state the respondent. Pain prevalence was 28% to 84% in studies that used self‐report only, 31% to 85% in studies that used both self‐ and proxy‐reports, and 58% in the single study that used proxy‐report only. Pain prevalence was 24% to 89% in studies that did not state the respondent.

**TABLE 1:** Prevalence estimates for different bodily locations.

Pain location	Range of prevalence estimates	Contributing studies
Head and trunk		
Head	13–26%	10, 32, 50, 71
Neck/cervical	21–63%	10, 24, 32, 47, 71, 72, 73
Back (including low back, upper back, spine, thoracic, or lumbosacral)	7–90%	10, 23, 24, 25, 26, 27, 32, 47, 50, 56, 71, 72, 74, 75, 76, 77
Abdomen/pelvis	12% or 14%	71, 77
Stomach	19%	10
Chest	5% or 11%	71, 77
Iliosacral	8%	23
Lower limb		
Lower limb (general)	8–70%	23, 24, 27, 47, 71, 74
Hip	13–49%	10, 23, 24, 32, 54, 71, 74, 77
Knee	23–39%	10, 23, 24, 32, 71
Upper limb		
Upper limb (general)	25–37%	27, 47, 74
Neck, shoulder, or arm	10–50%	10, 16, 23, 24, 32, 50, 71, 75, 77

The prevalence of chronic pain, defined as pain lasting longer than 3 months, was 75%^29^ and 78%.^30^ Three studies described prevalence of ‘chronic pain’, but used a different definition to pain lasting longer than 3 months. Prevalence of chronic pain, defined as daily pain for at least 1 year, was 24%^31^ and 82%,^32^ and prevalence of chronic pain (not defined) was 49%.^33^ Five studies reported the prevalence of distinct categories of pain intensity: very mild (10–13%), mild (11–21%), moderate (22–38%), severe (11–25%), or very severe (4%).^32^, ^34^, ^35^, ^36^, ^37^ In one study, 24% of adults reported severe or very severe pain in the previous 4 weeks.

#### Pain location

Eighteen studies reported prevalence by specific pain location and prevalence estimates are summarized in Table 1. Prevalence was highest in the neck, back, and lower limb. However, estimates varied considerably between studies.

#### Pain types

One study reported prevalence by pain type, with 34% of adults having nociplastic‐type pain, 11% having neuropathic‐type pain, and 16% having mixed nociplastic/neuropathic‐type pain.^38^

#### Comparison with adults without CP or with a reference population

Prevalence of pain was higher in adults with CP than in adults without CP, although certainty was low, derived from six studies (Table S10). The prevalence of back pain, lower limb pain, and upper limb pain was higher among adults with CP than age‐, sex‐, and body mass index‐matched adults with typical development (*p* < 0.05).^26^ There was a ‘significant difference’ in mean scores of bodily pain between adults with CP and a reference group.^32^ The prevalence of chronic pain was higher in adults with CP than the general population in two studies (24% vs. 15%, *p* = 0.01;^31^ and 75% vs. 39%, *p* < 0.001).^29^ The age‐adjusted prevalence of joint pain was 44% in adults with CP compared with 28% in adults without it (*p* < 0.001).^39^ One study found no difference in pain (score >3) between adults with CP and a reference group (*p* = 0.41).^40^

### Prognostic factors for pain

Twenty‐eight studies, including 4063 adults with CP, described prognostic factors for pain presence and intensity in adults with CP. Twenty‐one were cross‐sectional studies and six were cohort studies. Sample sizes ranged from 17 to 1591. Eight studies (29%) reported data on adults with CP living in the USA. The mean age ranged from 21.2 years to 42.3 years. The percentage of females in each sample ranged from 20% to 100% (median 49%). Thirteen studies (46%) included adults with CP in all GMFCS levels. Three studies (11%) included adults in GMFCS levels I to III only. Studies examined associations between 42 different prognostic factors and pain presence or pain intensity among adults with CP (Table S11). Two studies did not report any *p*‐values to indicate strength of evidence to support associations, and so findings are not reported in text.^41^, ^42^

#### Socio‐demographic factors

Effect estimates for associations between pain and socio‐demographic factors are presented in Table 2. Age was associated with pain prevalence in three studies: two reported higher prevalence with increasing age^32^, ^43^ and one reported a difference in the prevalence of pain across age groups with no consistent direction.^10^ Three studies found pain prevalence was higher in females than males^10^, ^35^, ^44^ while two reported no association between sex and pain prevalence.^29^, ^32^ There was no evidence that the prevalence of pain was associated with education,^32^, ^43^ employment status,^43^ or accommodation status.^43^

**TABLE 2:** Summary of findings: associations between socio‐demographic factors and pain among adults with cerebral palsy.

Prognostic factor	Outcome	Results	*n*	Study
Age	Pain presence^a^	Age associated with higher pain presence rho = 0.271, *p* < 0.05	70	İçağasıoğlu et al.^43^
	Pain presence^b^	Age associated with higher pain presence unadjusted OR 1.34, 95% CI 1.11–1.63, *p* < 0.01	398	Jahnsen et al.^32^
	Pain presence^a^	16–19 years, 64%; 20–24 years, 63%; 25–29 years, 65%; 30–39 years, 75%; 40–49 years, 70%; 50–76 years, 70%, *p* < 0.029	1591	Rodby‐Bousquet et al.^10^
Sex or gender	Pain presence^a^	Prevalence of pain higher in females than males adjusted OR 4.2, 95% CI 1.0–20.2, *p* = 0.049	61	Jacobson et al.^35^
	Pain presence^b^	No association; female vs. male unadjusted OR 1.32, 95% CI 0.85–2.05	398	Jahnsen et al.^32^
	Pain presence^a^	Pain presence higher in females than males; 74% vs. 61%, OR 1.65, 95% CI 1.32–2.06	1591	Rodby‐Bousquet et al.^10^
	Chronic pain presence^c^	Pain presence higher in females than males: neck pain (*p* < 0.05); shoulder pain (*p* < 0.05); arm pain (*p* < 0.05); back pain (*p* < 0.05); hip pain (*p* < 0.05); knee pain (*p* < 0.05); foot/ankle pain (*p* < 0.05)	149	Opheim et al.^44^
	Chronic pain presence^c^	No association (females vs. males) OR 2.83, 95% CI 0.67–11.88, *p* = 0.15	56	Van Der Slot et al.^29^
	Pain intensity^a^	Pain intensity higher in female than males *r* = 0.16 (*p*‐value not reported)	17	Chin et al.^41^
Education	Pain presence^a^	No association rho = −0.013, *p* = 0.848	70	İçağasıoğlu et al.^43^
	Pain presence^b^	No association (*p*‐value not reported)	398	Jahnsen et al.^32^
	Pain intensity^a^	Higher educational attainment associated with higher pain intensity *r* = 0.23 (*p*‐value not reported)	17	Chin et al.^41^
Accommodation	Pain presence^a^	No association, rho = 0.181, *p* = 0.134	70	İçağasıoğlu et al.^43^
Employment status	Pain presence^a^	No association, rho = −0.134, *p* = 0.270	70	İçağasıoğlu et al.^43^
Household income	Pain intensity^a^	Higher household income associated with lower pain intensity *r* = −0.19 (*p*‐value not reported)	17	Chin et al.^41^

Abbreviations: CI, confidence interval; OR, odds ratio.

^a^ Self‐ or proxy‐report.

^b^ Respondent not reported.

^c^ Self‐report.

#### Function, subtype, and impairment type

Effect estimates for associations between pain, function, CP subtype, and impairments are presented in Table 3. There is low certainty evidence from five studies (*n* = 1882) that the prevalence of pain does not differ across GMFCS levels (Table S12). Three studies found no association between GMFCS level and prevalence.^29^, ^34^, ^45^ In one study, prevalence was higher in GMFCS levels IV and V than in level I (OR 1.65, 95% CI 1.12–2.44 and OR 1.97, 95% CI 1.26–3.10).^10^ Similarly, one study found prevalence was lower in GMFCS levels I and II compared with GMFCS levels III to V (OR 0.18, *p* < 0.05).^46^ Pain intensity was not associated with GMFCS level^47^, ^48^ or wheelchair use.^49^

**TABLE 3:** Summary of findings: associations between factors related to cerebral palsy and pain among adults with cerebral palsy.

Prognostic factor	Outcome	Results	*n*	Study
Gross Motor Function Classification System level	Pain presence^a^	No association; Prevalence in levels I 62%, II 66%, III 67%, IV 69%, V 71%; *p* = 0.407	153	Jonsson et al.^34^
	Pain presence^b^	Higher prevalence in level IV vs. I and level V vs. I; Prevalence in levels 1, 66%; II, 66%; III, 67%; IV, 67%; V, 67% II vs. I OR 1.14, 95% CI 0.80–1.61 III vs. I OR 1.36, 95% CI 0.91–2.02 IV vs. I OR 1.65, 95% CI 1.12–2.44 V vs. I OR 1.97, 95% CI 1.26–3.10	1591	Rodby‐Bousquet et al.^10^
	Pain presence^c^	Higher prevalence in levels III–V vs. I–II; Prevalence in levels I–II vs. III–V: 5.8% vs. 32.7%; OR 0.18, *p* < 0.05	52	Park and Kim^46^
	Pain presence^b^	No association (*p* = 1.0)	30	Yamashita et al.^45^
	Chronic pain presence^a^	No association; OR 1.68, 95% CI 0.67–4.23, *p* = 0.47	56	Van Der Slot et al.^29^
	Pain magnitude^b^	No association; mean (SD) pain in level I, 1.31 (1.1); II, 1.37 (0.76); III, 1.30 (0.95); IV 1.20 (0.94), V 1.5 (1.1), *p* = 0.95	97	Sienko^48^
	Pain intensity^b^	Higher level associated with lower pain intensity *r* = −0.14 (*p*‐value not reported)	17	Chin et al.^41^
	Pain intensity^b^	No association	48	Sandström et al.^47^
Wheelchair use	Pain intensity^a^	Wheelchair users vs. non‐wheelchair users: no association	26	Malone and Vogtle^49^
Subtype or anatomical distribution	Pain presence^a^	Prevalence in unilateral spastic 57%; bilateral spastic 78%; dyskinetic 66%; ataxic 33%; *p* = 0.029	153	Jonsson et al.^34^
	Pain presence^b^	Prevalence in unilateral spastic 67%; bilateral spastic 68%; dyskinetic 65%; ataxic 57%; mixed type 66% *p* = 0.393	1591	Rodby‐Bousquet et al.^10^
	Chronic pain^a^	No difference in chronic hip pain between unilateral and bilateral CP, *p* = 0.05; difference in chronic foot/ankle pain between unilateral and bilateral CP, *p* = 0.026	149	Opheim et al.^44^
Communication Function Classification System level	Pain presence^a^	Prevalence in levels I, 70%; II, 69%; III, 50%; IV, 50%; V, 38%; *p* = 0.021	153	Jonsson et al.^34^
	Pain presence^b^	Prevalence in levels I, 73%; II, 63%; III, 57%; IV, 65%; V 61% II vs. I OR 0.56, 95% CI 0.39–0.79; III vs. I OR 0.44, 95% CI 0.29–0.65; IV vs. I OR 0.51, 95% CI 0.32–0.81; V vs. I OR 0.35, 95% CI 0.21–0.60	1591	Rodby‐Bousquet et al.^10^
	Pain intensity^b^	Higher level associated with lower pain intensity *r* = −0.21 (*p*‐value not reported)	17	Chin et al.^41^
Manual Ability Classification System level	Pain intensity^b^	Higher level associated with lower pain intensity *r* = −0.10 (*p*‐value not reported)	17	Chin et al.^41^
	Pain presence^b^	Prevalence in levels I, 68%; II, 68%; III, 63%; IV, 63%; V, 68%; *p* = 0.407	1591	Rodby‐Bousquet et al.^10^
	Pain presence^b^	No association (*p* = 1.0)	30	Yamashita et al.^45^
Eating and Drinking Ability Classification System	Pain presence^b^	Prevalence in levels I, 68%; II, 69%; III, 65%; IV, 63%; V, 70%; *p* = 0.734	1591	Rodby‐Bousquet et al.^10^
Intellectual disability	Pain presence^a^	Prevalence in those with intellectual disability (44%) vs. without intellectual disability (71%); *p* = 0.004	153	Jonsson et al.^34^
	Pain presence^a^	Association between intellectual disability and pain; *p* = 0.037	63	Turk et al.^50^
	Pain presence^b^	rho = 0.023, *p* = 0.848	70	İçağasıoğlu et al.^43^
	Pain intensity^b^	Presence of intellectual disability associated with lower pain intensity *r* = −0.12 (*p*‐value not reported)	17	Chin et al.^41^
Severity of disability	Pain presence^c^	Prevalence lower in those with moderate/significant disability compared with mild disability (*p* = 0.045)	37	Terjesen et al.^51^
Deterioration in walking function or use of wheelchair	Pain intensity^a^	Higher pain intensity among those who reported deterioration in walking function compared with those with improved/unchanged walking function over 7 years: mean (SD) 54.9 (23.2) vs. 39.9 (22.8), 95% CI ‐6.3 to −23.8, *p* = 0.001	149	Opheim et al.^31^
	Chronic pain presence^c^	Deterioration in locomotion skills/use of wheelchair associated with higher prevalence of chronic pain adjusted OR 3.28, 95% CI 1.59–6.77, *p* < 0.001	398	Jahnsen et al.^32^
Six‐minute walk test distance	Pain intensity^a^	Higher distance associated with lower pain intensity adjusted *β* = 0.5, 95% CI 0.1–1.0 m, *p* < 0.05	126	Maanum et al.^52^
Self‐ratings of spasms	Pain presence^b^	More frequent spasms associated with higher pain; adjusted *β* = 0.70, 95% CI 0.05–1.36, *p* = 0.035	47	Flanigan et al.^53^
Spasticity	Pain presence^b^	Greater spasticity associated with higher pain; adjusted *β* = 0.13, 95% CI 0.01–0.25, *p* = 0.040	47	Flanigan et al.^53^
	Pain presence^b^	Presence of severe spasticity not associated with presence of pain adjusted OR 1.2, 95% CI 0.3–5.3, *p* = 1.000	61	Jacobson et al.^35^
	Hip pain presence^d^	Prevalence of hip pain associated with knee spasticity Prevalence of hip pain in those with mild (32%), moderate (49%), and severe (73%) knee spasticity; *p* = 0.04	77	Noonan et al.^54^
Range of motion	Hip pain presence^d^	Presence of hip pain associated with reduced hip abduction Mean abduction in those with hip pain was 50° compared with 75° in those without hip pain; *p* = 0.01	77	Noonan et al.^54^
	Pain intensity^b^	ROM in shoulder, elbow, wrist, hip, knee, and ankle associated with pain intensity: *r* = 0.64–0.74, *p* < 0.0001 to *p* = 0.006	48	Sandström et al.^47^
Flexion contracture in at least one hip	Hip pain presence^d^	No association Prevalence of hip pain among those with a flexion contracture compared with those without a flexion contracture (85% vs. 51%, *p* = 0.07)	77	Noonan et al.^54^
Windswept hips	Hip pain presence^d^	Prevalence of pain higher in those with windswept hips than those without windswept hips (76% vs. 43%, *p* = 0.02)	77	Noonan et al.^54^
Hip dislocation and subluxation	Hip pain presence^d^	No association between hip dislocation/subluxation and pain *p* = 0.38	77	Noonan et al.^54^
Posture	Pain presence^c^	Pain not associated with asymmetric posture (*p*‐value not reported)	102	Rodby‐Bousquet et al.^42^
	Pain presence^b^	Low back pain associated with postural ability in sitting (*p* = 0.05) No association between low back pain and quality of posture in frontal plane (*p* > 0.05) or quality of posture in sagittal plane (*p* > 0.05)	30	Yamashita et al.^45^
Selective dorsal rhizotomy in childhood	Spinal pain presence^b^	No difference in spinal pain presence between selective dorsal rhizotomy and no rhizotomy group at age 20 years (*p* = 0.488) or 25 years (*p* = 0.282)	66	Lundkvist Josenby and Westbom^56^
Orthopaedic surgery	Hip pain^b^	Femoral derotation osteotomy at age 5–12 years less hip pain compared with non‐femoral derotation osteotomy group in adulthood (effect size *Q* = 0.58, 95% CI 0.51–0.99)	61	Boyer et al.^55^
	Pain intensity^b^	Higher number of orthopaedic surgeries associated with higher pain intensity *r* = 0.24 (*p*‐value not reported)	17	Chin et al.^41^
Use of tone‐altering medication	Pain intensity^b^	Current use of tone‐altering medication associated with lower pain intensity *r* = −0.16 (*p*‐value not reported)	17	Chin et al.^41^

Abbreviations: CI, confidence interval; OR, odds ratio; SD, standard deviation.

^a^ Self‐report.

^b^ Self‐ or proxy‐report.

^c^ Respondent not reported.

^d^ Proxy‐report.

There is moderate certainty in evidence from two studies of a higher prevalence of pain in lower CFCS levels (Table S13). In one study, prevalence of pain was 70%, 69%, 50%, 50%, and 38% in CFCS levels I to V respectively (*p* = 0.021).^34^ In a second study, prevalence of pain was lower in each of CFCS levels II (OR 0.56, 95% CI 0.39–0.79), III (OR 0.44, 95% CI 0.29–0.65), IV (OR 0.51, 95% CI 0.32–0.81), and V (OR 0.35, 95% CI 0.21–0.60) compared with level I.^10^

Prevalence of pain differed according to subtype of CP in one study, with prevalence highest in bilateral spastic CP,^34^ but two studies found no association.^10^, ^44^ Prevalence of pain was not associated with the Manual Ability Classification System (two studies^10^, ^45^) or Eating and Drinking Ability Classification System level (one study^10^). One study reported prevalence was lower in people with intellectual disability,^34^ one study reported an association with intellectual disability but did not state the direction,^50^ and one study found no association.^34^, ^43^, ^50^ Pain prevalence was lower in those with moderate/significant disability compared with mild disability.^51^ Pain prevalence was higher in those who reported deterioration in locomotion skills or use of wheelchair during their lifetime.^32^ Pain intensity was higher in those who reported deterioration in walking function over 7 years^31^ and recorded shorter distances on the six‐minute walk test.^52^

#### Musculoskeletal factors or interventions

Effect estimates for associations between pain and musculoskeletal factors or interventions are presented in Table 3. More frequent spasms and increased spasticity were associated with higher pain.^53^ Increased knee spasticity, reduced hip abduction, and windswept hips were associated with the presence of hip pain.^54^ Postural ability in sitting was associated with presence of low back pain but the direction was not reported.^45^ Range of motion in the shoulder, wrist, elbow, hip, knee, and ankle was associated with pain but the direction was not reported.^47^ Femoral derotation osteotomy between the ages of 5 years and 12 years was associated with less hip pain in adulthood compared with no femoral derotation osteotomy among adults with bilateral CP.^55^ Hip flexion contracture or hip dislocation/subluxation were not associated with presence of hip pain.^54^ Selective dorsal rhizotomy in childhood was not associated with spinal pain in adulthood.^56^

#### Quality of life and other health outcomes

Effect estimates for associations between pain and quality of life or health outcomes are presented in Table 4. Presence of fatigue was associated with higher prevalence (OR 2.26, 95% CI 1.08–4.72).^29^, ^32^ Fatigue severity was associated with higher pain intensity^40^ but not prevalence.^35^ Sleep issues were associated with higher pain intensity (*r* = 0.71, 95% CI 0.58–0.80)^40^ but not pain prevalence (OR 2.5, 95% CI 0.6–12.4).^35^ Physical role function, emotional role function, physical health‐related quality of life, life satisfaction, and physical activity were associated with pain prevalence but the direction of association was unclear.^32^, ^44^ Presence of severe pain was associated with lower health‐related quality of life, and increased pain intensity was positively associated with depressive symptoms (*r* = 0.30, 95% CI 0.12–0.49).^4^, ^40^ There was no association between pain prevalence and comorbidity^43^ or mental health‐related quality of life.^44^

**TABLE 4:** Summary of findings: evidence for associations between health, quality of life, participation, and pain among adults with cerebral palsy.

Prognostic factor	Outcome	Results	*n*	Reference
Comorbidity	Pain presence^a^	Rho = 0.021, *p* = 0.864	70	İçağasıoğlu et al.^43^
Fatigue	Pain presence^a^	Presence of fatigue not associated with presence of pain adjusted OR 2.5, 95% CI 0.6–12.4, *p* = 0.288	61	Jacobson et al.^35^
	Chronic pain presence^c^	Severity of fatigue associated with higher prevalence of chronic pain OR 2.26, 95% CI 1.08–4.72	56	Van Der Slot et al.^29^
	Chronic pain presence^b^	Fatigue associated with higher prevalence of chronic pain unadjusted OR 4.65, 2.69–8.05, *p* < 0.001	398	Jahnsen et al.^32^
	Pain intensity^c^	Fatigue associated with higher pain intensity *r* = 0.71, 95% CI 0.58–0.80	97	van Gorp et al.^40^
Sleep disturbances	Pain presence^a^	Sleep issues not associated with presence of pain adjusted OR 1.5, 95% CI 0.3–6.8, *p* = 0.736	61	Jacobson et al.^35^
	Pain intensity^c^	Sleep disturbances associated with higher pain intensity *r* = 0.41, 95% CI 0.24–0.58	97	van Gorp et al.^40^
Physical role function	Chronic pain presence^b^	Physical role function associated with lower prevalence of chronic pain adjusted OR 0.74, 95% CI 0.55–0.99, *p* < 0.05	398	Jahnsen et al.^32^
Physical subscale of SF‐36	Chronic pain presence^c^	Physical subscale of SF‐36 negatively associated with chronic pain presence, *r* = −0.34, *p* = 0.001	149	Opheim et al.^44^
Emotional role function	Chronic pain presence^b^	Emotional role function associated with lower chronic pain prevalence unadjusted OR 0.67, 95% CI 0.47–0.96, *p* < 0.05	398	Jahnsen et al.^32^
Mental subscale of SF‐36	Chronic pain presence^c^	No association *p* = 0.63	149	Opheim et al.^44^
Depressive symptoms	Pain intensity^c^	Pain intensity positively associated with depressive symptoms *r* = 0.30, 95% CI 0.12–0.49	97	van Gorp et al.^40^
Anxiety/depression symptoms	Pain intensity^a^	Positive association between anxiety/depression symptoms and pain intensity *r* = 0.10	17	Chin et al.^41^
Participation	Pain frequency^c^	Higher satisfaction level with participation associated with lower frequency of back pain (rho = −0.467, *p* = 0.012) Satisfaction level with participation not associated with frequency of upper limb pain (rho = −0.061, *p* = 0.760) Satisfaction level with participation not associated with frequency of lower limb pain (rho = −0.370, *p* = 0.052) Accomplishment level with participation not associated with frequency of back pain (rho = −0.339, *p* = 0.077) Accomplishment level with participation not associated with frequency of upper limb pain (rho = −0.168, *p* = 0.394) Accomplishment level with participation not associated with frequency of lower limb pain (rho = −0.233, *p* = 0.233)	28	du Toit et al.^26^
	Pain frequency^c^	Higher accomplishment level with participation associated with lower frequency of back pain (rho = −0.564, *p* = 0.001) Accomplishment level with participation not associated with frequency of upper limb pain (rho = −0.203, *p* = 0.282) Accomplishment level with participation not associated with frequency of lower limb pain (rho = −0.312, *p* = 0.093) Satisfaction level with participation not associated with frequency of back pain (rho = −0.338, *p* = 0.068) Satisfaction level with participation not associated with frequency of upper limb pain (rho = −173, *p* = 0.360) Satisfaction level with participation not associated with frequency of lower limb pain (rho = −246, *p* = 0.190)	30	Eken et al.^27^
Life satisfaction	Chronic pain presence^b^	Life satisfaction associated with higher prevalence of chronic pain adjusted: OR 2.65, 95% CI 1.31–5.38, *p* < 0.01	398	Jahnsen et al.^32^
Physical activity	Chronic pain presence^b^	Physical activity associated with higher prevalence of chronic pain unadjusted OR 1.90, 95% CI 1.21–2.99, *p* < 0.01	398	Jahnsen et al.^32^
Deterioration of skills	Chronic pain presence^b^	Deterioration of skills associated with higher prevalence of chronic pain adjusted OR 3.28, 95% CI 1.59–6.77, *p* < 0.001	398	Jahnsen et al.^32^
Health‐related quality of life	Severe pain presence^c^	Negative association between presence of severe pain and health‐related quality of life (*p* < 0.05)	408	Jarl et al.^36^
Catastrophizing symptoms	Pain intensity^a^	Catastrophizing symptoms associated with higher pain intensity (*r* = 0.31)	17	Chin et al.^41^
Self‐reported body image of the low back	Pain presence^a^	Higher levels of self‐reported body perception disturbance associated with presence of low back pain (*p* < 0.01)	30	Yamashita et al.^43^

Abbreviations: CI, confidence interval; OR, odds ratio.

^a^ Self‐ or proxy‐report.

^b^ Respondent not reported.

^c^ Self‐report.

#### Participation

Effect estimates for associations between pain and participation are presented in Table 4. One cohort study reported that a higher satisfaction level with participation, but not a higher accomplishment level, was associated with less frequent back pain (rho = −0.467, *p* = 0.012).^26^ Conversely, a cross‐sectional study found higher accomplishment with participation, but not higher satisfaction, was associated with less frequent back pain (rho = −0.564, *p* = 0.001).^27^ Accomplishment and satisfaction with participation were not associated with frequency of upper limb or lower limb pain.^26^, ^27^

### Pain assessment tools

Four studies, including 297 adults with CP, reported validity or reliability of self‐report tools to assess pain among adults with CP. No study reported feasibility of measures. Sample size ranged from 18 to 160. Two studies were conducted in the USA, one study was conducted in the Netherlands, and one in Israel. Mean age ranged from 36 to 40 years 7 months. Samples included 46% to 50% females. GMFCS level was not reported in any study.

The validity of 10 self‐reported pain measures was reported in four cross‐sectional studies: eight assessed pain intensity and two assessed pain interference among adults with CP. Associations and assessment of the certainty in the evidence are presented in Table 5. There is moderate certainty in the evidence for construct validity of the Pyramid Pain Scale for assessing pain intensity. Pain intensity rated on the Pyramid Pain Scale had an excellent correlation with pain stimulation intensity and a moderate correlation with the Facial Action Coding System among adults with and without intellectual disability.^19^ There is low certainty in the evidence for construct validity of the 11‐ and 21‐point numerical rating scales, 5‐ and 16‐point verbal rating scales, and 6‐ and 7‐point faces scale for assessing pain intensity. There were good to excellent associations between pain intensity measured on each tool, and poor to good associations between pain intensity on each tool and measures of depressive symptoms and pain interference, among adults with at most mild cognitive impairment.^57^ In this study 28% used a communication device. There is also low certainty in the evidence for construct validity of the Pain Assessment Instrument for CP for assessing pain intensity, with poor to good associations between self‐reported pain intensity, and physiotherapist‐ and caregiver‐reported pain intensity among adults with severe CP.^58^

**TABLE 5:** Summary of findings: psychometric properties of pain measures.

Reference	*n*	Construct	Pain measure; administration	Reference measure	Construct validity	Concurrent validity	Internal consistency	Certainty^a^
Jensen et al.^57^	69	Average pain intensity during previous 24 hours	NRS‐11; self‐reported using standardized interview protocol	Depressive symptoms (CES‐D); pain interference (modified version of pain interference scale of BPI)	NRS‐11 vs. BPI: *r* = 0.25*; NRS‐11 vs. CES‐D *r* = 0.30*	vs. NRS‐21 *r* = 0.87** vs. VRS‐5 *r* = 0.79** vs. VRS‐16 *r* = 0.69** vs. FS‐6 *r* = 0.71** vs. FS‐7 *r* = 0.71**	NR	Low (owing to methodological limitations and imprecision)
			NRS‐21; self‐reported using standardized interview protocol		NRS‐21 vs. BPI: *r* = 0.41**; NRS‐21 vs. CES‐D *r* = 0.36**	vs. NRS‐11 *r* = 0.87** vs. VRS‐5 *r* = 0.82** vs. VRS‐16 *r* = 0.84** vs. FS‐6 *r* = 0.83** vs. FS‐7 *r* = 0.81**	NR	Low (owing to methodological limitations and imprecision)
			VRS‐5; self‐reported using standardized interview protocol		VRS‐5 vs. BPI: *r* = 0.29*; VRS‐5 vs. CES‐D *r* = 0.23	vs. NRS‐21 *r* = 0.82** vs. NRS‐11 *r* = 0.79** vs. VRS‐16 *r* = 0.85** vs. FS‐6 *r* = 0.83** vs. FS‐7 *r* = 0.81**	NR	Low (owing to methodological limitations and imprecision)
			VRS‐16; self‐reported using standardized interview protocol		VRS‐16 vs. BPI: *r* = 0.42**; VRS‐16 vs. CES‐D *r* = 0.30*	vs. NRS‐11 *r* = 0.69** vs. NRS‐21 *r* = 0.84** vs. VRS‐5 *r* = 0.0.85** vs. FS‐6 *r* = 0.82** vs. FS‐7 *r* = 0.85**	NR	Low (owing to methodological limitations and imprecision)
			FS‐6; self‐reported using standardized interview protocol		FS‐6 vs. BPI: *r* = 0.38**; FS‐6 vs. CES‐D *r* = 0.33*	vs. NRS‐11 *r* = 0.71** vs. NRS‐21 *r* = 0.83** vs. VRS‐5 *r* = 0.79** vs. VRS‐16 *r* = 0.82** vs. FS‐7 *r* = 0.85**	NR	Low (owing to methodological limitations and imprecision)
			FS‐7; self‐reported using standardized interview protocol		FS‐7 vs. BPI: *r* = 0.50**; FS‐7 vs. CES‐D *r* = 0.38**	vs. NRS‐11 *r* = 0.59** vs. NRS‐21 *r* = 0.81** vs. VRS‐5 *r* = 0.77** vs. VRS‐16 *r* = 0.82** vs. FS‐6 *r* = 0.85**	NR	Low (owing to methodological limitations and imprecision)
Boldingh et al.^58^	160	Pain intensity and location in 21 situations	PAICP; self‐reported	Caregiver and physiotherapy scores on PAICP in usually painful situations	vs. physiotherapist *r* = −0.03 to *r* = 0.15 vs. caregiver *r* = 0.06–0.20	NR	Cronbach α = 0.83 (95% CI 0.77–0.87)	Low (owing to methodological limitations and imprecision)
				Caregiver and physiotherapy scores on PAICP in usually not painful situations	vs. physiotherapist *r* = −0.03 to *r* = 0.20 vs. caregiver *r* = −0.01 to *r* = 0.35**	NR	Cronbach α = 0.65 (95% CI 0.55–0.73)	Low (owing to methodological limitations and imprecision)
				Caregiver and physiotherapy scores on PAICP in possibly painful situations	vs. physiotherapist *r* = 0.29** to *r* = 0.52** vs. caregiver *r* = 0.23** to *r* = 0.48**	NR	Cronbach α = 0.81 (95% CI 0.75–0.86)	Low (owing to methodological limitations and imprecision)
Benromano et al.^19^	18	Pain intensity	Pyramid Pain Scale; self‐reported with or without assistance	Facial expressions to noxious stimuli assessed using the Facial Action Coding System	vs. pain stimulation intensity (with intellectual disability): *r* = 0.63 *p* < 0.0001 (without intellectual disability): *r* = 0.83 *p* < 0.0001	vs. Facial Action Coding System (with intellectual disability): *r* = 0.49, *p* < 0.01	NR	Moderate (owing to imprecision)
Tyler et al.^59^	50	Pain interference	Chronic Pain Grade; self‐reported	Average pain intensity over previous week (NRS‐11)	Chronic Pain Grade vs. NRS‐11 *r* = 0.16*	NR	Cronbach α = 0.59	Low (owing to methodological limitations and imprecision)
			BPI; self‐reported	Average pain intensity over previous 24 hours (NRS‐11)	BPI composite score vs. NRS‐11 *r* = 0.66* 10 interference items on BPI vs. NRS *r* = 0.33* to *r* = 0.68*	NR	Cronbach α = 0.89	Low (owing to methodological limitations and imprecision)

Abbreviations: BPI, Brief Pain Inventory; CES‐D, Centre for Epidemiological Studies Depression Scale; CI, confidence interval; FS‐6, 6‐point Faces Scale; FS‐7, 7‐point Faces Scale; NRS‐11, 11‐Point Numeric Rating Scale; NRS‐21, 21 point (0–100) numerical rating scale; PAICP, Pain Assessment Instrument for Cerebral Palsy; VRS‐5, 5‐point verbal rating scale; VRS‐16, 16‐point verbal rating scale.

^a^ Construct validity comparing index test against reference measure.

* *p* < 0.05;

** *p* < 0.01.

There is low certainty in the evidence for construct validity of the Chronic Pain Grade and Brief Pain Inventory for assessing pain interference. Associations between self‐reported pain interference using the Chronic Pain Grade and average pain intensity over the previous week were poor.^59^ There was a good association between pain interference on the Brief Pain Inventory and average pain intensity over 1 week.^59^ Internal consistency was good to excellent for the Pain Assessment Instrument for CP, moderate for the Chronic Pain Grade, and excellent for the Brief Pain Inventory.^58^, ^59^

### Effectiveness and safety of interventions for pain

Five studies, including 143 adults with CP, evaluated the effectiveness of an intervention to reduce pain in adults with CP (Table 6). Four were RCTs and one was an uncontrolled pre−/post‐intervention design. Sample size ranged from 13 to 57. Studies were conducted in Sweden, Spain, the Netherlands, France, and Korea. Mean age ranged from 20 to 46 years. Samples included 10% to 63% females. Two studies included adults with CP classified in all GMFCS levels, two included adults in GMFCS levels I to IV, and one did not report GMFCS level. Three studies assessed pain intensity using a numerical rating scale or visual analogue scale, one assessed bodily pain intensity and interference using the 36‐Item Short Form Survey (SF‐36), and one assessed pressure pain. Two studies stated pain was self‐reported, and two did not state the respondent.

**TABLE 6:** Summary of findings: interventions to reduce pain among adults with cerebral palsy.

Reference	*n* ^a^	Intervention	Comparator	Outcomes^b^	Effect	Adverse events
Jacobson et al.^60^	16	Botulinum neurotoxin A; one session of electromyographically guided intramuscular injections of BoNT‐A (Dysport, 100 U/mL, up to a maximal total dose of 1500 U); dose median (range) 9.2 mL (6.6–15); 920 U (660–1500); number of injections: median (range) 13 (8–24); number of muscles: median (range) 2.5 (1–4)	Placebo; one session of electromyographically guided intramuscular injections or normal saline; dose: median (range) 10.2 mL (4–13.9); number of injections: median (range) 15 (8–24); number of muscles: median (range) 4 (1–4)	Short‐term (6 weeks) Treatment responders, defined as a reduction of pain intensity of two or more steps on the NRS	Between‐group difference: effect estimate not reported, *p* = 1.00	Intervention group: mild pain and discomfort during and immediately after the injections (*n* = 5, 75%); transient focal weakness in treated muscles (*n* = 2, 38%). Comparison group: lymphoma (*n* = 1, 13%)
				Short‐term (6 weeks) Pain intensity on NRS; self‐reported	Between‐group mean difference: 2.0, 95% CI −0.60–4.60, *p* = 0.121	
Riquelme et al.^63^	32	Somatosensory therapy in addition to standardized physical therapy for 12 weeks; four types of somatosensory task focused on face and hands: touch, proprioception, vibration, and stereognosis; task difficulty was increased from the first to the second weekly session; two 45‐minute weekly sessions	Standardized physical therapy	Short‐ and intermediate‐term (0 months and 3 months) Pressure pain measured with a digital dynamometer and using a flat rubber tip	Effect estimate not reported; ‘significant group × time × body interaction effect’, *p* < 0.05	Not reported
Slaman et al.^62^	57	Active lifestyle and sports participation intervention for 6 months; counselling on daily physical activity and sedentary behaviour guided by a personal coach, supervised and home‐based training physical fitness training, counselling on sports participation	Usual care	Short‐and long‐term (0 months and 6 months) Bodily pain intensity and interference measured with SF‐36; self‐reported	Short‐term Between‐group mean difference: 5.47 (95% CI −7.12 to 18.06) Long‐term Between‐group mean difference: 15.14 (95% CI 3.44–26.85)	Not reported
Vidailhet et al.^64^	13	Bilateral pallidal stimulation; leads were implanted bilaterally at one session while the patients were under general anaesthesia, in accordance with institutional protocols at each centre	No comparator^c^	Long‐term (1 year) Pain intensity on a visual analogue scale (0–10); respondent not reported	Pre–post mean difference: effect estimate not reported, *p* = 0.33	Eight participants had adverse events: spontaneous stimulator arrest owing to exposure to an external magnetic field (*n* = 1); cervical myelopathy (*n* = 1); sub‐clavicular pain (*n* = 1); type of adverse event not reported (*n* = 5)
Yi et al.^61^	16	Botulinum neurotoxin A into neck muscles; mean (SD) dose 139.7 U (50.5) (minimum–maximum 40–200); mean number of muscles injected = 3	Placebo; saline injection	Short‐ and intermediate‐term (4 weeks and 12 weeks) Pain intensity on NRS; respondent not reported TWSTRS pain subscale; respondent not reported	Short‐term NRS: Between‐group difference; effect estimate not reported, *p* = 0.06 TWSTRS pain subscale: between‐group difference in change; effect estimate not reported, *p* = 0.0013 Intermediate‐term NRS: between‐group difference: effect estimate not reported, *p* = 0.18 TWSTRS pain subscale: Between‐group difference in change; effect estimate not reported, *p* = 0.020	Intervention group: dysphagia (*n* = 2) Comparison group: dysphagia (*n* = 1)

Abbreviations: CI, confidence interval; NRS, numerical rating scale; TWSTRS, Toronto Western Spasmodic Torticollis Rating Scale.

^a^ Number included in comparison.

^b^ Short‐ and intermediate‐term outcomes reported as time post‐intervention.

^c^ Pre−/post‐intervention study.

#### Interventions

Two studies examined the effect of botulinum neurotoxin A (BoNT‐A) on pain intensity compared with placebo. One study, of 16 adults with chronic pain related to spasticity, examined the effect of one session of electromyographically guided intramuscular injections of BoNT‐A on pain intensity at 6 weeks post‐intervention. The other examined the effect of one session of BoNT‐A into neck muscles at 4 weeks and 12 weeks post‐intervention on pain intensity in 16 adults with dyskinesia and cervical dystonia. One study examined the effect of a 6‐month active lifestyle and sports participation intervention on pain intensity and interference, compared with usual care, among 57 young adults, immediately following the intervention and 6 months post‐intervention. The intervention consisted of counselling on daily physical activity and sedentary behaviour guided by a personal coach to discuss barriers and facilitators of physical behaviour; physical fitness training, consisting of supervised centre and home‐based training and focused on increasing cardiopulmonary fitness and muscle strength; and counselling on sports participation to find suitable, accessible, and appropriate sports and sports facilities in the person's day‐to‐day environment. One study examined the effect of 12 weeks of somatosensory therapy in addition to standardized physical therapy compared with standardized physical therapy on pressure pain, among 32 adults without chronic pain, immediately following the intervention and 3 months post‐intervention. The final study examined the effect of bilateral pallidal stimulation, with leads implanted bilaterally at one session while the patients were under general anaesthesia, on pain intensity among 13 adults with disabling dystonia at 12 months post‐intervention.

#### Effectiveness

There is low certainty evidence from two studies of no effect of BoNT‐A on pain intensity compared with placebo in the short‐term or intermediate‐term (Table S14). There was no difference in pain intensity, on the numerical rating scale (range 0–10), among adults with chronic pain related to spasticity who received BoNT‐A, compared with placebo, at 6 weeks post‐intervention (between‐group mean difference − 2.0, 95% CI −0.60 to 4.60, *p* = 0.121).^60^ There was also no difference in the percentage of responders between groups (defined as a reduction in pain intensity of two or more steps on a numerical rating scale). There was also no difference in pain intensity on the numerical rating scale among adults with dyskinesia and cervical dystonia who received BoNT‐A into neck muscles compared with placebo at 4 weeks (effect estimate not reported, *p* = 0.06) or 12 weeks post‐intervention (effect estimate not reported, *p* = 0.18).^61^ There was a difference in change in the pain subscale of the Toronto Western Spasmodic Torticollis Rating Scale between groups at 4 weeks (*p* = 0.0013) and 12 weeks post‐intervention (*p* = 0.0200) (effect estimate not reported).

There is evidence from one study for an effect of a 6‐month active lifestyle and sports participation intervention on pain in the long‐term but not in the short‐term, compared with usual care (low certainty evidence; Table S15). Pain, as measured on the SF‐36 (range 0–100), was not different between groups immediately post‐intervention (between‐group mean difference 5.47 [95% CI −7.12 to 18.06]) but was lower in the intervention group at 6 months post‐intervention (between‐group mean difference 15.14 [95% CI 3.44–26.85]).^62^

There is evidence from one study for an effect of somatosensory therapy in addition to standardized physical therapy on pain in the short‐term, compared with standardized physical therapy (low certainty evidence; Table S15). Pressure pain thresholds measured with a digital dynamometer reduced following somatosensory therapy, compared with standardized physical therapy (‘group × time × body interaction effect’, *p* < 0.05), although the mean difference between groups at follow‐up was not reported.^63^

There was no evidence for an effect of bilateral pallidal stimulation on pain among adults with disabling dystonia in the long‐term (very low certainty; Table S16). There was no difference in pain intensity on a visual analogue scale (0–10) at 12 months following bilateral pallidal stimulation compared with baseline pain intensity (mean difference not reported, *p* = 0.33).^64^

#### Adverse events

In one study of BoNT‐A for adults with chronic pain related to spasticity, five (75%) reported mild pain and discomfort during and immediately after the injections and two (38%) reported transient focal weakness; one adult (13%) who received placebo developed lymphoma.^60^ In a second study of BoNT‐A for adults with dyskinesia and cervical dystonia, two (25%) in the intervention group and one in the comparison group (16%) developed dysphagia. Adverse events were not reported in studies investigating a 6‐month active lifestyle and sports participation intervention or 12 weeks of somatosensory therapy in addition to standardized physical therapy. Eight participants had adverse events following bilateral pallidal stimulation, including ‘spontaneous stimulator arrest’ due to exposure to an external magnetic field (*n* = 1), cervical myelopathy (*n* = 1), and sub‐clavicular pain (*n* = 1).

## DISCUSSION

This review synthesizes the evidence relating to the epidemiology and characteristics of pain, prognostic factors for pain, pain assessment tools, and interventions for pain among adults with CP. There was evidence that the prevalence of pain was higher in adults with CP than in those without CP (low certainty). There was evidence that the prevalence of pain is higher among adults with better communication function, as measured by the CFCS (moderate certainty), and no evidence that the prevalence of pain differs across GMFCS levels (low certainty). There was evidence that numerical, verbal, and pictorial rating scales are valid for assessing pain intensity in adults with CP (low to moderate certainty). There was no evidence that BoNT‐A improves pain intensity among adults with spasticity or dyskinesia and cervical dystonia, or that bilateral pallidal stimulation improves pain intensity among adults with disabling dystonia (very low to low certainty). There was evidence that a 6‐month active lifestyle and sports intervention may reduce pain in the long‐term and that 12 weeks of somatosensory therapy may reduce pain in the short‐term (low certainty).

A large proportion of adults with CP experience pain, although estimates ranged from 24% to 89%. At least 75% of adults experience chronic pain defined as lasting longer than 3 months. Back, neck, and lower limb pain were most prevalent, although there was large variation in the prevalence of pain at each body site. Although adults with CP are more likely to experience pain, only three studies allowed direct comparison with the prevalence in adults without CP. In these studies, the prevalence of chronic pain was 36% higher in adults with CP, the prevalence of daily pain for at least 1 year was 9% higher in adults with CP, and the age‐adjusted prevalence of joint pain was 18% higher in adults with CP.^29^, ^31^, ^39^ Only one study described pain type, with nociplastic pain being more common than neuropathic pain.^38^

To our knowledge, this is the largest review of pain prevalence in adults with CP. Despite including a substantially greater number of studies than previous reviews that reported pain prevalence, in part because of differences in eligibility criteria, findings were largely in agreement. In a meta‐analysis of individual participant data from 14 samples of adults with CP aged at least 18 years, collected from 2000 to 2016, pain prevalence in adults with CP was 70% (95% CI 62–78).^16^ Despite differences in eligibility criteria, the range of pain prevalence estimates, from 38% to 89%, was similar to the current review (24%–89%). A systematic review of children and young adults with CP (2–23 years) reported pain prevalence in eight studies ranged from 14% to 76%.^2^ This review reported prevalence of leg pain ranged from 32% to 82% and back pain ranged from 9% to 25%, suggesting that back pain is less prevalent in children and young adults with CP than in adults with CP. A meta‐analysis indicated that leg pain was most prevalent in adults with CP followed by back pain.^29^ However, estimates of prevalence by pain site were not comparable with this review because they were calculated as a percentage of individuals with pain rather than prevalence in the total population.

Although 29 studies examined 42 different prognostic factors for pain in adults with CP, a relatively small number of studies investigated the same factor. GMFCS level was the most frequently investigated factor (five studies), with mixed findings.^10^, ^29^, ^34^, ^45^, ^46^ The only consistent evidence for prognostic factors exists for communication, with those having better communication being more likely to report pain. However, this could potentially indicate an underestimation of pain in adults with less efficient communication and those with complex communication needs who may not be able to report their pain. Some studies indicated that the prevalence of pain increases with age and is higher in females with CP than in males.^10^, ^32^, ^35^, ^43^, ^44^ Currently, findings from studies in this review suggest that CP subtype, upper limb function, eating and drinking function, and presence of intellectual disability are not prognostic factors for prevalent pain.

There was some agreement between findings from this and previous reviews about prognostic factors for pain. This and previous reviews of children and adults with CP indicate that females are more likely to have pain than males.^2^, ^16^ There is mixed evidence from previous reviews about the association between age and pain prevalence.^2^, ^16^ Pain prevalence did not differ across CP subtype in this or a previous review.^16^ Although a meta‐analysis of individual participant data found pain prevalence was higher in those classified in GMFCS levels II and IV than in level I,^16^ a review of children and young adults found inconsistent evidence from individual studies, as we did.^2^

The findings indicate the Pyramid Pain Scale, 11‐point and 21‐point numerical rating scales, 5‐point and 16‐point verbal rating scales, 6‐point and 7‐point faces scale, and the Pain Assessment Instrument for CP are valid tools for assessing pain intensity in adults with CP with and without intellectual disability. The Chronic Pain Grade and Brief Pain Inventory are also valid and reliable for assessing pain interference in adults with CP. In all studies, adults with CP with and without intellectual disability self‐reported pain. In two studies, adults used communication devices to enable them to complete the self‐report tool. This indicates that these tools are appropriate for use in these subgroups of adults with CP and should be used where possible instead of proxy‐reports.

Only five studies examined the effectiveness of interventions to reduce pain in adults with CP. There is no evidence at present that BoNT‐A or bilateral pallidal stimulation reduce pain. An active lifestyle and sports intervention may reduce pain in the long‐term, by 15 points on a 0 to 100 scale. Somatosensory therapy may also reduce pain compared with standard physical therapy; however, the size of the effect following the intervention was not reported and a clinically relevant outcome measure was not used. Reporting of adverse events was incomplete in all studies and inconsistent across studies.

This is the first review to investigate the effectiveness of interventions to reduce pain in adults with CP. A review of pain management for children with CP in 2018 found most evidence available related to management of procedural pain or postoperative pain, with limited evidence on interventions to reduce chronic pain in people with CP.^17^ There was mixed evidence relating to the effect of BoNT‐A, compared with placebo, on pain related to hypertonia.^17^ However, evidence from the single RCT comparing BoNT‐A with placebo found no effect.^17^ The review identified evidence to support intrathecal baclofen therapy for pain secondary to hypertonia, clown‐care therapy for procedural pain during BoNT‐A injections, and pharmacological interventions to improve postoperative pain. We did not identify any studies examining effectiveness of these interventions in adults with CP.

The evidence base on pain among adults with CP is incomplete. Most studies identified addressed the first objective of this review, to describe the prevalence and incidence of pain among adults with CP. Similar to a previous review of pain in adults with CP,^16^ this review found lack of a standardized method for assessing pain, which probably contributes to the large variation in prevalence estimates. The presence of pain was also elucidated using a range of self‐reported and/or proxy‐reported questions or scales that varied in terms of their assessment of severity, duration, and frequency of pain. In the included studies, adults with CP or proxies were asked to report current pain, pain in the previous 3 months, pain in previous week, recurrent pain, persistent pain, problematic pain, pain in previous 4 weeks, daily pain for 1 year or more, mild pain, or moderate or severe pain. According to the International Association for the Study of Pain, chronic pain is ‘pain which has persisted beyond normal tissue healing time’ which, in the absence of other factors, is generally taken to be 3 months.^65^ Studies included in this review defined chronic pain as pain lasting more than 3 months, daily pain lasting for at least 1 year or they did not define chronic pain.^29^, ^30^, ^31^, ^32^, ^33^ At a minimum, the International Association for the Study of Pain definition of chronic pain should be adopted in studies of adults with CP and consensus should be achieved on a standardized question to accurately identify adults with CP with current pain and chronic pain for research and clinical purposes.

Further, studies used self‐reported pain, proxy‐reported pain, or both, with most studies not reporting respondent type. There was no clear pattern that pain prevalence was higher when self‐ or proxy‐reports were used. There was also no clear pattern that the association between factors and pain differed across respondent type. Future studies must report respondent type and use self‐report where possible. We did identify tools that are valid in people with intellectual disability and communication impairments that may be used in future studies, although the number of tools assessed and number of studies examining psychometric properties is limited. It is also important that future validation studies describe intellectual disability and communication ability in the sample to enable researchers and clinicians to select appropriate tools for their population.

The evidence base on prognostic factors for pain is seriously limited by a lack of cohort studies examining factors that predict incident pain. As well as the limitation of cross‐sectional studies for determining causality, many studies included in the review did not report the direction of association between prognostic factors and pain. We were unable to conclude whether associations between prognostic factors such as GMFCS level and pain were in part due to respondent type, communication impairment, or intellectual disability on the basis of the totality of the evidence. It is essential that future studies control for these potential confounding factors in the design or analysis; at present, only 5 of the 28 studies examining prognostic factors used strategies for dealing with confounding factors.

Despite the potential impact of pain on quality of life, psychological outcomes, and employment among adults with CP,^6^, ^12^, ^13^ there is incomplete evidence about the safety and effectiveness of interventions to reduce pain in adults with CP. Although the five included studies aimed to reduce pain or pain intensity, the target population differed in terms of subtype of CP and presence of chronic pain, limiting the applicability of findings to subgroups of adults with CP. Further, no study aimed to address a specific pain mechanism. Future studies need to develop theory‐ and evidence‐informed interventions to target specific pain mechanisms to increase the likelihood of effectiveness.

All studies examining the effectiveness of interventions had serious methodological limitations. Although four were RCTs, random sequence generation was unclear in three and allocation was not concealed or allocation concealment was unclear in all four. Estimates of intervention effect are often exaggerated in trials with inadequate or unclear sequence generation or allocation concealment,^63^ particularly in trials with self‐report outcome measures.^66^, ^67^ In addition, four studies had sample sizes of fewer than 50 participants and one had 57 participants, which may have contributed to the lack of evidence of an effect because of lower statistical power, or conversely the small sample size may have inflated effect sizes.^68^, ^69^, ^70^

An extensive search of databases and hand‐searching of reference lists of related reviews was conducted. However, grey literature was not searched. Conference abstracts were also excluded. A broad definition of pain was used, which probably resulted in variation in findings between studies and challenges with interpreting the evidence. Owing to the broad scope of the review, we did not include studies reporting pain interference or pain coping, which are important areas for further research in adults with CP. Studies of any adults with CP were included, regardless of subtype, age, and type of impairment, which resulted in samples in many studies not being comparable with each other. A meta‐analysis was not conducted to pool data for any objective. Although there were sufficient data to pool estimates of pain prevalence, there was substantial clinical heterogeneity between studies, namely differences between samples, definitions of pain, and methods to assess pain, which limits the use of a pooled prevalence estimate. A conservative approach to assessing overall risk of bias for included studies was taken (i.e. ‘the worst score counts’ principle). However, all studies had at least two items rated as ‘no’ or ‘unclear’. Finally, the broad scope of the review and methodological diversity required to address each question represented challenges for evidence synthesis. However, we used a recommended guideline for each review type required to address each question to ensure rigour.

## CONCLUSION

This review synthesizes current evidence on pain in adults with CP, considering four important interrelated issues: the burden of pain, predictors of pain, how to identify pain, and how to reduce pain in adults with CP. These questions need to be collectively addressed to support clinicians to better manage pain in this population. Many adults with CP experience pain, and pain is more prevalent in adults with CP than in adults without CP, although prevalence estimates vary considerably between studies. Despite this, evidence for prognostic factors for pain is inconsistent and methodologically flawed. The quality of evidence for prognostic factors and interventions is very low to low. Well‐designed cohort studies investigating prognostic factors for the development of pain patterns in adults with CP are needed to design evidence‐based and theory‐based interventions and better inform treatment recommendations and individual patient management. Further, there is a need for large, high‐quality, well‐reported RCTs that assess the effectiveness of established multidisciplinary approaches to the management of pain among adults with CP.

## FUNDING INFORMATION

This project was supported by a grant from the Cerebral Palsy Foundation.

## CONFLICT OF INTEREST STATEMENT

The authors have stated that they had no interests that might be perceived as posing a conflict or bias.

## Supporting information


**Appendix S1:** Search strategy for PubMed.

**Appendix S2:** Eligibility criteria by question.

**Appendix S3:** GRADE criteria.

**Table S1:** Description of included studies.

**Table S2:** Quality appraisal of prevalence studies reporting prevalence of pain.

**Table S3:** Quality appraisal of cross‐sectional studies comparing pain prevalence between adults with and without cerebral palsy.

**Table S4:** Quality appraisal of cross‐sectional studies examining prognostic factors for pain.

**Table S5:** Quality appraisal of cohort studies examining prognostic factors for pain.

**Table S6:** Quality appraisal of studies examining psychometric properties of pain assessment tools.

**Table S7:** Quality appraisal of randomized controlled trials examining effectiveness of interventions.

**Table S8:** Quality appraisal of quasi‐experimental studies examining effectiveness of interventions.

**Table S9:** Summary of findings: prevalence of pain among adults with cerebral palsy.

**Table S10:** Summary of clinical evidence profile for comparison: adults with cerebral palsy compared to adults without cerebral palsy.

**Table S11:** Description of prognostic factors for pain examined among adults with cerebral palsy.

**Table S12:** 11 Summary of clinical evidence profile for comparison 2: GMFCS levels I–V.

**Table S13:** Summary of clinical evidence profile for comparison: CFCS levels I–V.

**Table S14:** Summary of clinical evidence profile for comparison: pharmacological intervention compared to placebo.

**Table S15:** Summary of clinical evidence profile comparison: non‐pharmacological intervention compared to no intervention or usual care.

**Table S16:** Summary of clinical evidence profile comparison: surgical intervention compared to no intervention or usual care.

**Figure S1:** Flow diagram.

## Data Availability

No data available.
