# Supplementary material for: Pain in adults with cerebral palsy: A systematic review
Source: Dev Med Child Neurol. 2025 Feb 12;67(7):854–74. doi: 10.1111/dmcn.16254 (PMC12134420; doi:10.1111/dmcn.16254)
Supplement: Supplementary file 1 — Appendix S1: Search strategy for PubMed. [file DMCN-67-854-s019.docx]

**Appendix 1 Search strategy for PubMed**

| # | Search Strategy |
| --- | --- |
| 1 | "cerebral palsy" OR "Cerebral Palsy"[MeSH] OR "cerebral paresis"[tiab] |
| 2 | **"Pain"[MeSH] OR "Pain Management"[MeSH] OR "Pain Perception"[MeSH] OR "Pain Measurement"[MeSH] OR "Analgesia"[MeSH] OR pain[tiab] OR pains[tiab] OR ache[tiab] OR aches[tiab] OR "physical suffering"[tiab] OR "intense discomfort"[tiab] OR "unpleasant sensation"[tiab] OR "unpleasant sensations"[tiab] OR "unpleasant sensory experience*"[tiab] OR nociception[tiab] OR "nociceptive neurons"[tiab] OR arthralgia[tiab] OR angina[tiab] OR anginas[tiab] OR earache[tiab] OR earaches[tiab] OR toothache[tiab] OR toothaches[tiab] OR headache[tiab] OR headaches[tiab] OR mastodynia[tiab] OR mastodynias[tiab] OR mastalgia[tiab] OR mastalgias[tiab] OR metatarsalgia[tiab] OR metatarsalgias[tiab] OR "morton neuroma"[tiab] OR myalgia[tiab] OR myalgias[tiab] OR neuralgia[tiab] OR neuralgias[tiab] OR neurodynia[tiab] OR "piriformis muscle syndrome"[tiab] OR "sciatic nerve"[tiab] OR "sciatic nerves"[tiab] OR "sciatic neuropathy"[tiab] OR sciatica[tiab] OR "renal colic"[tiab] OR stenocardia[tiab] OR odontalgia[tiab] OR glossalgia[tiab] OR glossodynia[tiab] OR "slit ventricle syndrome"[tiab] OR "muscle soreness"[tiab] OR "muscle tenderness"[tiab] OR "pubic symphysis dysfunction"[tiab] OR "symphysis pubic dysfunction"[tiab] OR causalgia[tiab] OR allodynia[tiab] OR "Schnitzler syndrome"[tiab] OR "burning sensation"[tiab] OR cystalgia[tiab] OR dysmenorrhea[tiab] OR dyspareunia[tiab] OR dysuria[tiab] OR hyperalgesia[tiab] OR hyper-algesia[tiab] OR hyperalgia[tiab] OR hypoalgesia[tiab] OR hypo-algesia[tiab] OR hypoalgia[tiab] OR hypalgesia[tiab] OR mittelschmerz[tiab] OR backache[tiab] OR backaches[tiab] OR myodynia[tiab] OR "painful limbs"[tiab] OR "painful limb"[tiab] OR "painful moving extremities"[tiab] OR "painful moving extremity"[tiab] OR "painful leg"[tiab] OR "painful legs"[tiab] OR "painful arm"[tiab] OR "painful arms"[tiab] OR "painful hands"[tiab] OR "painful hand"[tiab] OR odynophagia[tiab] OR vulvodynia[tiab] OR orchialgia[tiab] OR orchiodynia[tiab] OR testalgia[tiab] OR "biliary colic"[tiab] OR "kidney colic"[tiab] OR antinociception[tiab] OR analgesia[tiab] OR analgesic[tiab] OR algometry[tiab]** |
| 3 | #1 AND #2 |
| 4 | #3 NOT (Allchild[Filter] NOT Alladult[Filter]) |
| 5 | "Guideline"[pt] OR "practice guideline"[pt] OR "consensus development conference"[pt] OR "consensus development conference, NIH"[pt] OR guideline*[ti] OR standards[ti] OR consensus*[ti] OR recommendat*[ti] OR guideline*[cn] OR standards[cn] OR consensus*[cn] OR recommendat*[cn] OR "practice parameter*"[ti] OR "position statement*"[ti] OR "practice bulletin*"[ti] OR "policy statement*"[ti] OR CPG[ti] OR CPGs[ti] OR "best practice*"[ti] OR (care[ti] AND (path[ti] OR paths[ti] OR pathway[ti] OR pathways[ti] OR map[ti] OR maps[ti] OR plan[ti] OR plans[ti] OR standard[ti])) OR ((critical[ti] OR clinical[ti] OR practice[ti]) AND (path[ti] OR paths[ti] OR pathway[ti] OR pathways[ti] OR protocol*[ti])) OR (algorithm*[ti] AND (pharmacotherap*[ti] OR chemotherap*[ti] OR chemotreatment*[ti] OR therap*[ti] OR treatment*[ti] OR intervention*[ti])) OR (algorithm*[ti] AND (screening[ti] OR examination[ti] OR test[ti] OR tested[ti] OR testing[ti] OR assessment*[ti] OR diagnosis[ti] OR diagnoses[ti] OR diagnosed[ti] OR diagnosing[ti])) OR guideline*[ot] OR standards[ot] OR consensus*[ot] OR recommendat*[ot] OR "practice parameter*"[ot] OR "position statement*"[ot] OR "practice bulletin*"[ot] OR "policy statement*"[ot] OR CPG[ot] OR CPGs[ot] OR "best practice*"[ot] OR (care[ot] AND (path[ot] OR paths[ot] OR pathway[ot] OR pathways[ot] OR map[ot] OR maps[ot] OR plan[ot] OR plans[ot] OR standard[ot])) OR ((critical[ot] OR clinical[ot] OR practice[ot]) AND (path[ot] OR paths[ot] OR pathway[ot] OR pathways[ot] OR protocol*[ot])) OR (algorithm*[ot] AND (pharmacotherap*[ot] OR chemotherap*[ot] OR chemotreatment*[ot] OR therap*[ot] OR treatment*[ot] OR intervention*[ot])) OR (algorithm*[ot] AND (screening[ot] OR examination[ot] OR test[ot] OR tested[ot] OR testing[ot] OR assessment*[ot] OR diagnosis[ot] OR diagnoses[ot] OR diagnosed[ot] OR diagnosing[ot])) OR (("Systematic review"[ti] OR "systematic review"[pt] OR "systematic review"[ot]) AND ("practice guideline*"[tiab] OR "treatment guideline*"[tiab] OR "clinical guideline*"[tiab] OR "guideline recommendation*"[tiab])) |
| 6 | "systematic"[filter] OR "meta-analysis"[pt] OR "meta-analysis as topic"[mh] OR "meta analy*"[tw] OR metanaly*[tw] OR metaanaly*[tw] OR "met analy*"[tw] OR "integrative research"[tiab] OR "integrative review*"[tiab] OR "integrative overview*"[tiab] OR "research integration*"[tiab] OR "research overview*"[tiab] OR "collaborative review*"[tiab] OR "collaborative overview*"[tiab] OR "systematic review"[pt] OR "systematic reviews as topic"[mh] OR "systematic review*"[tiab] OR "technology assessment*"[tiab] OR "technology overview*"[tiab] OR "technology appraisal*"[tiab] OR "Technology Assessment, Biomedical"[mh] OR HTA[tiab] OR HTAs[tiab] OR "comparative efficacy"[tiab] OR "comparative effectiveness"[tiab] OR "outcomes research"[tiab] OR "indirect comparison*"[tiab] OR "Bayesian comparison"[tiab] OR (("indirect treatment"[tiab] OR "mixed-treatment"[tiab]) AND comparison*[tiab]) OR Embase*[tiab] OR Cinahl*[tiab] OR "systematic overview*"[tiab] OR "methodological overview*"[tiab] OR "methodologic overview*"[tiab] OR "methodological review*"[tiab] OR "methodologic review*"[tiab] OR "quantitative review*"[tiab] OR "quantitative overview*"[tiab] OR "quantitative synthes*"[tiab] OR "pooled analy*"[tiab] OR Cochrane[tiab] OR Medline[tiab] OR Pubmed[tiab] OR Medlars[tiab] OR handsearch*[tiab] OR "hand search*"[tiab] OR "meta-regression*"[tiab] OR metaregression*[tiab] OR "data synthes*"[tiab] OR "data extraction"[tiab] OR "data abstraction*"[tiab] OR "mantel haenszel"[tiab] OR peto[tiab] OR "der-simonian"[tiab] OR dersimonian[tiab] OR "fixed effect*"[tiab] OR "multiple treatment comparison"[tiab] OR "mixed treatment meta-analys*"[tiab] OR "umbrella review*"[tiab] OR (("multiple paramet*"[tiab]) AND ("evidence synthesis"[tiab])) OR (("multi-paramet*"[tiab]) AND ("evidence synthesis"[tiab])) OR ((multiparameter*[tiab]) AND ("evidence synthesis"[tiab])) OR "Cochrane Database Syst Rev"[Journal] OR "health technology assessment winchester, england"[Journal] OR "Evid Rep Technol Assess (Full Rep)"[Journal] OR "Evid Rep Technol Assess (Summ)"[Journal] OR "Int J Technol Assess Health Care"[Journal] OR "GMS Health Technol Assess"[Journal] OR "Health Technol Assess (Rockv)"[Journal] OR "Health Technol Assess Rep"[Journal] |
| 7 | (clinical[tiab] AND trial[tiab]) OR "Clinical Trials as Topic"[MeSH] OR "Clinical Trial"[Publication Type] OR random*[tiab] OR "Random Allocation"[MeSH] OR "Therapeutic Use"[Subheading] OR "Multicenter Study"[Publication Type] OR "Multicenter Studies as Topic"[MeSH] OR "controlled trial*"[tiab] OR ((single[tiab] OR doubl*[tiab] OR tripl*[tiab] OR treb*[tiab]) AND (blind*[tiab] OR mask*[tiab])) |
| 8 | "Cohort Studies"[MeSH] OR "Cross Sectional Studies"[MeSH] OR cohort[tiab] OR longitudinal[tiab] OR prospective[tiab] OR retrospective[tiab] OR observational[tiab] OR "cross sectional"[tiab] OR "crosssectional"[tiab] OR "prevalence stud*"[tiab] |
| 9 | #5 OR #6 OR #7 OR #8 |
| 10 | #4 AND #9 |
| 11 | #10 AND (1990/1/1:3000/12/12[pdat]) |
