# Supplementary material for: Pain in adults with cerebral palsy: A systematic review
Source: Dev Med Child Neurol. 2025 Feb 12;67(7):854–74. doi: 10.1111/dmcn.16254 (PMC12134420; doi:10.1111/dmcn.16254)
Supplement: Supplementary file 2 — Appendix S2: Eligibility criteria by question. [file DMCN-67-854-s017.docx]

**Appendix 2: Eligibility criteria by question**

| **1. What is the prevalence and incidence of pain among adults with cerebral palsy?** | |
| --- | --- |
| **Condition** | Prevalence or incidence of pain |
| **Context** | Any country worldwide and any setting (e.g. population-based or hospital-based). |
| **Population** | Adults with CP aged 16 years or older. However, where studies include people aged 16 and 17 years, they must also include adults aged 18 years and older to be included in the review. |
| **Study design** | Cohort studies  Cross-sectional studies  Systematic reviews that directly address our question of interest and were conducted in the last 3 years |

| **2. What are prognostic factors for pain presence and intensity in adults with CP?** | |
| --- | --- |
| **Population** | Adults with CP aged 16 years or older. However, where studies include people aged 16 and 17 years, they must also include adults aged 18 years and older to be included in the review. |
| **Exposure** | Any modifiable or non-modifiable socio-demographic or clinical factor, whose association with pain is examined, such as gender, GMFCS level, musculoskeletal complications. |
| **Outcome** | - Prevalent or incident pain - Pain intensity |
| **Study design** | Cohort studies  Case-control studies  Cross-sectional studies  Systematic reviews that directly address our question of interest and were conducted in the last 3 years |

| **3. What are the psychometric properties and feasibility of tools to assess pain among adults with CP?** | |
| --- | --- |
| **Population** | Adults with CP aged 16 years or older. However, where studies include people aged 16 and 17 years, they must also include adults aged 18 years and older to be included in the review. |
| **Instrument** | Patient-reported and clinician-reported instruments |
| **Construct** | - Pain presence - Pain intensity - Pain location - Pain interference |
| **Outcome** | - Validity - Reliability - Responsiveness - Feasibility |
| **Study design** | Any quantitative study design that aims to develop and/or assess the psychometric properties of an instrument  Systematic reviews that directly address our question of interest and were conducted in the last 3 years |

| **4. Are interventions for reducing pain, directly or indirectly through targeting prognostic factors, in adults with CP safe and effective?** | |
| --- | --- |
| **Population** | Adults with CP aged 16 years or older. However, where studies include people aged 16 and 17 years, they must also include adults aged 18 years and older to be included in the review. |
| **Intervention** | Any intervention that aims to effect prognostic factors for pain or pain. Interventions may include, but not be limited to, pharmacological interventions, surgical interventions, and physical or psychological interventions. Interventions may be used to address pain secondary to hypertonia or musculoskeletal complications, pain due to procedures, and post-operative pain. |
| **Comparator** | - Usual care - No intervention - A modified version of the intervention - Different intervention - Placebo |
| **Outcome** | - Pain presence - Pain intensity - Pain duration or frequency - Adverse events |
| **Study design** | RCTs  controlled before- and after-studies  uncontrolled before- and after-studies  interrupted time series  Systematic reviews that directly address our question of interest and were conducted in the last 3 years |
