# Supplementary material for: Pain in adults with cerebral palsy: A systematic review
Source: Dev Med Child Neurol. 2025 Feb 12;67(7):854–74. doi: 10.1111/dmcn.16254 (PMC12134420; doi:10.1111/dmcn.16254)
Supplement: Supplementary file 3 — Appendix S3: GRADE criteria. [file DMCN-67-854-s010.docx]

For prognostic factors for pain, observational studies started with high certainty ratings.^1^ For intervention effectiveness, RCTs started with high certainty ratings. For the comparison of an index test to a reference method, all studies were initially judged as high regardless of the study design.^2^

To ensure consistency of GRADE judgements we applied the following assessment criteria:

1. Serious study limitations: we downgraded once if more than 25% of the participants were from studies classified as being at overall high risk of bias
2. Inconsistency: we downgraded once if the studies did not show effects in the same direction.
3. Indirectness: we downgraded once if more than 50% of the participants were outside the target group.
4. Imprecision: we downgraded once if there were fewer than 400 participants for continuous data and fewer than 300 events for dichotomous data.
5. Publication bias: we downgraded once where there was direct evidence of publication bias.

We will consider single studies to be inconsistent and imprecise, unless more than 400 participants were randomised for continuous outcomes or more than 300 for dichotomous outcomes.

^1^Foroutan F, Guyatt G, Zuk V, Vandvik PO, Alba AC, Mustafa R et al. GRADE Guidelines 28: Use of GRADE for the assessment of evidence about prognostic factors: rating certainty in identification of groups of patients with different absolute risks. J Clin Epidemiol 2020;121:62-70.

^2^Yang B, Mustafa RA, Bossuyt PM, Brozek J, Hultcrantx M, Leeflang MMG et al. GRADE Guidance: 31. Assessing the certainty across a body of evidence for comparative test accuracy. J Clin Epidemiol 2021;136:146-156.
