# Supplementary material for: Pain in adults with cerebral palsy: A systematic review
Source: Dev Med Child Neurol. 2025 Feb 12;67(7):854–74. doi: 10.1111/dmcn.16254 (PMC12134420; doi:10.1111/dmcn.16254)
Supplement: Supplementary file 4 — Table S1: Description of included studies. [file DMCN-67-854-s003.docx]

**Supplemental table 1 Description of included studies**

| Author (year) | Study design | Country | Years completed | Population (source and eligibility) | Sample size | Age, yr, mean (SD) [min, max] | Female, % | CP subtype, % | GMFCS level,^b^ % | ID, % | Objective^c^ |
| --- | --- | --- | --- | --- | --- | --- | --- | --- | --- | --- | --- |
| Asuman et al.^13^ | cohort | Sweden | 1990-2015 | All individuals diag- nosed with CP ICD10 code G80 (ICD9: 343) in the National Patient Register (NPR), the Medical Birth Register, and the Swedish CP- Follow up Program and national register (CPUP) between 1990 and 2015 were identified. Information was linked to this population on labor outcomes and social security benefits from the Longitu- dinal Integrated Database for Health Insurance and Labour Market Studies (LISA), demographics from the Register of the Total Popu- lation, health care utilization and medical diagnoses from NPR, and dispensed pain medications from the National Prescribed Drug Register for the years 2006-2015; included aged 20-64 yr living in Sweden. Excluded adults who (i) had no CP-diagnosis after the age of three, (ii) acquired a brain injury after the age of 2 years without a CP- diagnosis before the acquired brain damage, (iii) had other diagnoses of the central or peripheral nervous system as well as progressive diseases, muscle disease and metabolic diseases that are incompatible with a CP-diagnosis, and (iv) those who had been excluded in the CPUP register. | 6899 | 40.8 | 46% | NR | NR | NR | Prevalence |
| Benner et a;.^78^ | cross-sectional | Netherlands | NR | Adults known to a rehabilitation centre in the Hague with a regional adherence area; included those born between 1965 and 1974 | 49 | 39.8 (3.0) [35-45] | 45% | spastic unilateral 38%; spastic bilateral 38%; non spastic 25% | I-III 80%; IV-V 20% | 22% | prevalence |
| Bourelle et al.^20^ | cross-sectional | France | 2004 | Between 1976 and 1981, 23 children underwent an extra-articular subtalar arthrodesis for severe valgus hindfoot. From this group, 17 (26 feet) were reviewed retrospectively. | 17 | NR | NR | quadriplegic 41%; spastic diplegic 47%; hemiplegic 12% | NR | NR | prevalence |
| Dauvergne et al.^79^ | cross-sectional | France | 2007 | Beneficiaries of the three main health insurance plans who benefited from an exemption from the ticket moderator for long-term conditions for infantile cerebral palsy, coded G80 in the international classification of diseases tenth version (ICD10); aged ≥16 years and residing in Brittany | 562 | 36^a^ | 44% | NR | wheelchair user 47% | 76% | prevalence |
| de Albuquerque et al.^80^ | cross-sectional | Brazil | 2017 | Conducted at Association of Parents and Friends of Exceptional Children known as APAE in Brazil of the cities of Tubarão and Orleans, State of Santa Catarina, Brazil; included GMFCS levels IV-V. Excluded adults with diagnosis of severe CP and those whose parents or caregivers could not understand the content of the consent form. | 93 | 23 [1-57] | 44% | NR | IV 47%; V 53% | NR | prevalence |
| Engel et al.^77^ | cross-sectional | USA | NR | Recruited from medical and rehabilitation clinics at the University of Washington Medical Center and from local residential and community-based treatment facilities for persons with developmental disabilities; included primary diagnosis of CP; documented IQ at least 70 or clinical judgement of health care provider or interviewer of no more than mild cognitive impairment; aged 18 or older. | 100 | 41 (12.2) [19-71] | 45% | Spastic 65%; mixed 24%; athetoid 5%; ataxic 4%; hypotonic 2% | Non-ambulatory 82% | NR | prevalence |
| Frank and De Souza ^21^ | cross-sectional | UK | NR | A specialist NHS service for the provision of electric powered wheelchairs; included aged ≥19 yr, community living electric powered indoor/outdoor wheelchair users. | 79 | 32.6 [19-70] | 47% | NR | wheelchair user 100% | NR | prevalence |
| Gallien  Et al.^22^ | cross-sectional | France | NR | Included aged ≥16 yr. | 562 | 36 (14) | 43% | NR | 0 5%; I 13%; II 17%; III 19%; IV 22%; V 21% | NR | prevalence |
| Garca Jalon et al.^81^ | cross-sectional | Northern Ireland | NR | Data from the Northern Ireland CP Register (NICPR) linked to the primary care registration records from the National Health Application and Infrastructure Service (NHAIS), and to the Enhanced Prescribing Database (EPD); included all individuals born between 1981 and 2008 (i.e. aged 4–31y in 2012), registered within NHAIS, and alive between 2010 and 2014. | 742 | [18-31] | NR | NR | NR | NR | prevalence |
| Gotze et al.^82^ | cross-sectional | Germany | 2022 | Adults attending a single centre specialised in the conservative and surgical treatment of children and adults with CP; included those who were 17 years when received multilevel surgery, with at least 7 years follow-up, bilateral CP, GMFCS levels I-III | 24 | 38 [24-60] | 42% | bilateral spastic 100% | I 8%; level II 62%; level III 30% | NR | prevalence |
| Hilberink et al.^75^ | cross-sectional | Netherlands | 2007 | Rehabilitation centre in The Hague, a centre with a regional function, covering the city of The Hague and the surrounding area; included adults born between 1 January 1965 and 31 December 1974 (aged 25-36 years at follow-up). | 54 | 30.0 (3.4) | 52% | NR | I 28%; II 34%; III 7%; IV 24%; V 7% | NR | prevalence |
| Hirsh et al.^71^ | cross-sectional | USA | NR | Adults who had participated in previous studies conducted by the research group and through a website and flyer posted in University medical clinics; included those who scored 17 or higher (or 14 or higher if using communication device) out of 25 on the Modified Mini-Mental Status Exam | 83 | 40.3 (13.6) [18-74] | 55% | spastic 47%; mixed 29%; athetoid 16%; ataxic 1%; hypotonic 1% | wheelchair user 76% | NR | prevalence |
| Hung et al.^73^ | cross-sectional | USA | NR | People who had office visits at the  departments of orthopaedics and/or physical medicine and  rehabilitation for any treatment; ≥18 years | 32 | 54.5 (10.5) [18-78] | 66% | spastic 78%; dyskinetic 6%; dyskinetic/ spastic mixed 3%; ataxic/spastic mixed 0%; Nonspecified 13% | I 25%; II 25%; III 22%; IV 19%; V 9% | NR | prevalence |
| Langerak et al.^24^ | cross-sectional | Germany | 2020 | Included adults with spastic diplegia who received their first orthopaedic intervention more than 15 years ago and treated  with an Interval Surgery Approach (ISA). Excluded adults  with neuromuscular disorders or another type of CP | 30 | 32.8 (28.1-39.3)^a^ | 60% | NR | I 50%; II 37%; III 13% | NR | prevalence |
| Murphy et al.^72^ | cross-sectional | USA | NR | Adults identified via United Cerebral Palsy  Association Affiliate of Alameda-Contra  Costa counties. | 101 | 42.6 [19-74] | 48% | spastic CP 48%; dyskinetic CP 52% | NR | NR | prevalence |
| Patatoukas et al.^76^ | cross-sectional | Greece | NR | NR | 33 | 29.6 (7.4) | 18% | NR | Wheelchair user 45% | NR | prevalence |
| Peterson et al.^38^ | cross-sectional | USA | 2012-2020 | Data collected prospectively from the Michigan Genomics Initiative and the analgesic Outcomes Study in University of Michigan; adults with CP aged ≥18 yr identified with ICD-9CM or ICD-10-CM code for a CP diagnosis | 71 | 39.3 (16.2) | 61% | NR | NR | NR | prevalence |
| Peterson et al.^39^ | cross-sectional | USA | 2002-2010 | Medical Expenditure Panel Survey (MEPS), an ongoing nationally representative survey of the US civilian, non-institutionalised population conducted annually by the Agency for Healthcare Research and Quality | 1015 | 58.2 (95% CI 56.9-59.6) | 34% | NR | NR | NR | prevalence |
| Salie et al.^74^ | cross-sectional | South Africa | NR | Adults with CP were recruited from databases of previous studies as well as referrals, word of mouth recommendations, and social media; included 23-40 years, excluded diagnosis of any neuromuscular disorder(s), inability to communicate and living outside a 100 km radius from Cape Town. | 30 | 34.8 (30.2-36.1)^a^ | 67% | spastic hemiplegia 7%; spastic diplegia 73%; spastic quadriplegia 10%; ataxia 3%; athetoid 7% | I 20%; II 20%; III 17%; IV 23%; V 20% | NR | prevalence |
| Schmidt et a.^14^ | cross-sectional | France and Germany | NR | Adults from French-German  TRANS-DISAB study, which focused on the impact of the  physical, social and attitudinal environment on participation, mental health, and QoL in emerging adults with  CP in the transition phase from late adolescence to adulthood (19–28 years). The TRANS-DISAB study is part  of the SPARCLE 3 cohort study, the third  wave of a longitudinal multicenter European observational population-based study. In SPARCLE 3, the  original SPARCLE population was extended by an additional cross-sectional sample recruited by health professionals who either approached the person directly or via their legal representative; included aged 19-28 years living in France or Germany | 198 | 23.5 (2.1) [19-28] | 46% | NR | I 37%; II 12%; III 14%; IV 22%; V 15% | NR | prevalence |
| Shrader et al.^33^ | cross-sectional | USA | NR | Adults identified from a historical database from the authors’ institution; included 25-45 years, GMFCS levels I-III | 126 | Self-reported group 29.7 (4.3); proxy-reported group 29.7 (4.1) | 44% | NR | self-reporting group: I 28%; II 47%; III 28%;  proxy-reporting group: I 10%; II 68%; III 22% | NR | prevalence |
| Tedroff et al.^28^ | cross-sectional | Sweden | NR | The study was undertaken at the Karolinska University Hospital between 2011 and 2012, 15-19 years after participants received SDR; included adults with spastic diplegia who underwent SDR at an average age of 4 years 7 months at the hospital between 1993 and 1997 | 18 | 22^a^ [20-27] | 22% | NR | I 17%; II 28%; III 17%; IV 33%; V 6% | NR | prevalence |
| Thorpe et al.^84^ | cross-sectional | USA | 2011-2014 | 20% sample of Medicare fee-for-service claim; included Medicare beneficiaries 65 years and older with CP identified by searching the inpatient, skilled nursing facility, home health, outpatient, carrier, and durable medical equipment claims for CP-related ICD-9-CM diagnostic codes covering all subtypes of CP. Included individuals if they had continuous Medicare Part A and Part B coverage for at least one full calendar year. | 9,060 | 65-69 yr 46%; 70-74 yr 22%; 75-79 yr 15%; 80-84 yr 10%; 85+ yr 8% | 55% | NR | NR | NR | prevalence |
| Veerbeek et al.^25^ | cohort | South Africa | 2021 | Included those with spastic diplegia (with mild unilateral upper extremity involvement allowed), who underwent SDR at  the Red Cross War Memorial Children’s Hospital in Cape  Town between 1981 and 1991. Excluded those with nonrelated CP injuries and/or who developed a neuromuscular disorder that may have resulted in and/or influenced their spinal abnormalities | 25 | 35.9 (34.3-41.5)^a^ | NR | NR | I 52%; II 36%; III 12% | NR | prevalence |
| Vidart et al.^6^ | cross-sectional | France, Germany, Sweden, Italy | 2018-2020 | Third wave of SPARCLE, a multicenter European observational  population-based cohort study. The SPARCLE cohort randomly sampled children with CP (aged 8–12 years in 2004–2005, SPARCLE1) from  population-based registers in eight European regions with an  overrepresentation of the most severe cases and from several independent sources in an additional region. SPARCLE3 included people in SPARCLE recruited from five European  regions, namely, southwest and southeast  France (departments of Haute-Garonne and Isère), northwest  Germany, western Sweden (Göteborg region), and central Italy  (Viterbo area) and an additional cross-sectional sample; included aged 22–27. | 164 | 24.3 (1.6) [22-27] | NR | spastic 76%, dyskinetic 17%, and ataxic 7%. | I-II 49%; III 18%; IV-V 32% | 52% | prevalence |
| Whitney et al.^12^ | cross-sectional | USA | Oct 2014 to Sept  2015 | Data from from the Optum Clinformatics Data Mart Database, a US nationwide deidentified single private payer administrative claims database. | 5888 | NR [18-64] | 47% | NR | NR | NR | prevalence |
| du Toit et al.^26^ | cohort | South Africa | NR | Recruited from a database composed at a school for children with special needs in Cape Town (South Africa); included those with spastic diplegia, GMFCS levels I-III, who received orthopedic interventions following the interval surgery approach. Excluded those with diagnosis of dystonia, athetosis, ataxia or hypotonia, a neurosurgical intervention such as a SDR, and living outside a 100 km radius from the testing facilities in Cape Town. | 28 | 39.0 (34.0-45.7)^a^ | 57% | NR | I 39%; II 43%; III 18% | NR | prevalence, prognostic |
| Eken et al.^27^ | cross-sectional | South Africa | NR | Recruited from a database of a  special school for disabled children in Cape Town, South Africa; included those with spastic diplegia, GMFCS levels I-III, received first orthopaedic surgery related to CP >15 years ago. Excluded those with diagnosis of dystonia, athetosis, ataxia or hypotonia, and received a neurosurgical intervention such as a SDR. | 30 | 32.8 (28.1-39.3)^a^ | 40% | NR | I 50%; II 37%; III 13% | NR | prevalence, prognostic |
| Flanigan et al.^53^ | cross-sectional | USA | NR | Rehabilitation clinics at a centre; Included aged 16-89 yr. Excluded those with other concurrent severe neurological or medical diagnoses that were likely to contribute to the participant’s functional impairment, participants with major limb amputations, or pregnant or lactating women. | 47 | 35.5 (14.5) [18-77] | 57% | NR | I 15%; II 19%; III 19%; IV 32%; V 15% | NR | prevalence, prognostic |
| İçağasıoğlu et al.^43^ | cross-sectional | Turkey | NR | Patients admitted to Istanbul Medeniyet University Göztepe Training and Research Hospital, Physical Medicine and Rehabilitation outpatient clinic; included aged ≥18 yr. | 70 | 29.4 (10.2) [19-68] | 47% | diplegic 24%; hemiplegic 21%; tetraplegic 33%; dyskinetic 16%; mixed 6% | I 9%; II 31%; III 17%; IV 17%; V 26% | 61% | prevalence, prognostic |
| Jacobson et al.^35^ | cross-sectional | Sweden | 2019 | Local registry including all children and young adults diagnosed with an International Classification of Diseases, 10th Revision code corresponding to CP and living in the greater Stockholm area; included aged 21 yr. | 61 | 21.2 (0.7) [20-22] | 44% | spastic bilateral 48%; spastic unilateral 36%; dyskinetic 11%; ataxic 3%; unclassifiable 2% | I 4%; II 50%; III 14%; IV 67%; V 100% | NR | prevalence, prognostic |
| Jahnsen et al.^32^ | cross-sectional | Norway | 2004 | From different eligible sources, such as the archives of relevant hospitals and institutions and from the Norwegian CP-association; included aged ≥18 yr, living in Norway. Excluded those with ID. | 406 | 34 (11.4) [18-72] | 49% | Hemiplegia 38%; diplegia 36%; dyskinesia 17%; quadriplegia 8% | NR | NR | prevalence, prognostic |
| Jarl et al.^4^ | cross-sectional | Sweden | NR | Data from the Swedish national quality registry and secondary prevention follow-up pro- gram for individuals with CP called Cerebral Palsy Follow-Up Program (CPUP). | 446 | 27 (10) [18-73] | 45% | spastic bilateral 52%; spastic unilateral 25%; dyskinetic 9%; ataxic 4%; non-classified & mixed 3% | I 30%; II 27%; III 18%; IV 15%; V 8%; missing 3% | NR | prevalence, prognostic |
| Jonsson et al.^34^ | cross-sectional | Sweden | NR | Adults in the CP register of western Sweden, born 1959-1978, and still residing in the Region of Vastra Gotaland; adults who had moved into the area were invited through patient organisations and habilitation units. | 153 | 48.3 (42-55)^a^ [37-58] | 43% | unilateral spastic 41%; bilateral spastic 36%; dyskinetic 19%; ataxic 4% | I 39%; II 21%; III 12%; IV 17%; V 11% | 22% | Prevalence, prognostic |
| Lundkvist and Westbom^56^ | cohort | Sweden | 2020 | CPUP registry; included those with spastic diplegia born 1990-2006. | NR |  | NR | spastic diplegia 100% | II-IV 100% | NR | prevalence, prognostic |
| Noonan et al.^54^ | cross-sectional | USA | 2004 | Six residential centers; included aged ≥21 yr, severely affected. All had severe muscle spasticity and contracture that precluded good walking ability, and the majority lacked head and trunk control and/or had severe mental retardation. | 77 | 40 [21-81] | 58% | spastic quadriplegia 86%; spastic diplegia 8%; mixed spastic-athetoid 5%; athetoid 1% | unable to walk 85%; able to walk with assistance 15% | NR | prevalence, prognostic |
| Opheim et al.^31^ | cohort | Norway | 2009 | A multidimensional questionnaire was mailed to 226 people who participated in a 1999 survey; included those with spastic CP, no ID, in GMFCS levels I-IV. | 149 | 40.0 (10.7) [24-76] | 49% | spastic bilateral 46%; unilateral 54% | I 46%; II 24%; III 15%; IV 13%; V 2% | NR | prevalence, prognostic |
| Opheim et al.^44^ | cross-sectional | Norway | NR | A multidimensional questionnaire was mailed to 226 people who participated in a 1999 survey; included those >18 yr, with spastic CP, no ID, in GMFCS levels I-IV. | 149 | 40.0 (10.7) | 49% | spastic bilateral 46%; unilateral 54% | I 46%; II 24%; III 15%; IV 13%; V 2% | NR | prevalence, prognostic |
| Park and Kim^46^ | cross-sectional | Korea | 2017 | Sampling of the resident area was based on a registry of disabled individuals covered by the Welfare of Disabled Persons Act in Korea; included those >18 yr, in GMFCS levels I-V, registered as having a brain lesion, diagnosed with CP by a doctor. | 53 | 32.2 (14.1) | NR | NR | NR | NR | prevalence, prognostic |
| Rodby-Bousquet et al.^10^ | cross-sectional | Sweden | 2020 | Data from all adults in the Swedish Cerebral Palsy Follow-up Program (CPUP) from 1st January 2015 to 31st December 2018. | 1591 | 25^a^ [16-76] | 45% | spastic unilateral 22%; spastic bilateral 55%; ataxic 5%; dyskinetic 14%; mixed type/unclassifiable 5% | I 22%; II 21%; III 15%; IV 19%; V 23% | NR | prevalence, prognostic |
| Rodby-Bousquet et al.^42^ | cross-sectional | Sweden | 2013 | Data from the CPUP health care program for adults in the south of Sweden; included those born between 1988 and 1991, living in the two southernmost counties of Sweden on the 1st of January 2009, examined from the start of October 2009 until the end of December 2011 (aged 18-21 yr). | 102 | NR [19-23] | 38% | unilateral spastic 25%; bilateral spastic 44%; dyskinetic 19%; ataxic 12%; unclassified 0% | I 37%;  II 21%;  III 13%;  IV 10%;  V 20% | NR | prevalence, prognostic |
| Sandstrom et al.^47^ | cross-sectional | Sweden | 2004 | Medical records from the paediatric rehabilitation centres where adults had been registered as children and adolescents; included aged ≥20 yr, living in a Swedish county. | 48 | 32.9 (8.2) | 52% | hemiplegia 27%; diplegia 40%; tetraplegia 19%; dyskinesia 15% | I 31%; II 23%; III 17%; IV 15%; V 15% | NR | prevalence, prognostic |
| Sarmiento et al.^30^ | cross-sectional | USA | 2019-2022 | Community Registry hosted on CP Research Network’s website gathers data directly from individuals with CP enrolled on the website; included ≥18 yr, able to understand English, able to consent. | 263^d^ | 43 (14) [18-74] | 75% | Spastic 67%; ataxic 5%; athetoid/chorea/dystonia 5%; hypotonic 1%; mixed 11%; unknown 11% | I 22%; II 50%; III 11%; IV 11%; V 3% | NR | prevalence, prognostic |
| Sienko^48^ | cross-sectional | USA | 2018 | Databases of the Shriners Hospitals for Children-Portland and the Child Development and Rehabilitation Center, Portland; included aged 18-30 yr, last known address in Oregon or Washington. Excluded those living in a foster home. | 97 | 23.8 (3.6) | 52% | NR | I 33%; II 20%; III 12%; IV 16%; V 19% | NR | prevalence, prognostic |
| Terjesen et al.^51^ | cross-sectional | Norway | NR | Diagnostic register for children who had been examined in the period 1968-73 at Berg Gård (then the central institution for cerebral palsy); included adults who now lived in Oslo or less than 200 km from Oslo. | 37 | 39 [27-62] | 51% | NR | cannot walk or can walk with support 49% | NR | prevalence, prognostic |
| Turk et al.^50^ | cross-sectional | USA | 1997 | Included those aged ≥20 yr, female only, community residents. | 63 | 37.7 (12.7) | 100% | spastic diplegia 29%; dyskinesia/posturing 25%; spastic hemiplegia 18%; other (ataxia, hypotonia, mixed) 16%; spastic quadriplegia 11% | wheelchair user 46% | 34% | prevalence, prognostic |
| Van Der Slot et al.^29^ | cross-sectional | Netherlands | 2012 | Ten rehabilitation centres in the western and central regions of the Netherlands including registers of paediatric rehabilitation, and through the Association of Physically Disabled Persons and their Parents; included aged 25-45 yr, spastic bilateral CP. Excluded those with severe cognitive impairment and those with full dependence on electric wheelchair. | 56 | 36.5 (5.8) | 38% | NR | I 23%; II 50%; III 20%; IV 7% | NR | prevalence, prognostic |
| van Gorp et al.^1^ | cross-sectional | Netherlands | 2021 | Former participants of the Pediatric Rehabilitation Research in the Netherlands program that recruited cohorts of children with CP between 2002 and 2007; included aged 21–34 yr. Excluded those with additional disorders affecting motor functioning or having an ID, which was classified as having attended special education for children with ID. | 97 | 28.5 (3.8) [21.6-34.3] | 39% | unilateral spastic 40%; bilateral spastic 45%; non-spastic 15% | I 51%; II 31%; III 5%; IV 10%; V 3% | NR | prevalence, prognostic |
| Boyer et al.^55^ | cohort | USA | NR | Gait laboratory database; included those aged ≥25 yr with bilateral CP, FDO-group included those with: only one proximal external FDO performed between ages 5–12 years (10–90th percentile for historic patients), implants removed, no pelvic osteotomy, acceptable knee varus-valgus range of motion, pre- operative gait analysis (baseline) <18 months prior to the FDO or a gait analysis at which individuals met all other criteria. Non-FDO group was matched on baseline anteversion and mean stance hip rotation (i.e., averaged over the stance phase of gait). | 61 | FDO group: 27.8 (2.6) [25.1-35.4]; no-FDO group 28.9 (3.5) [25.0-35.6] | FDO group 50%; no-FDO group 64% | FDO group: spastic 92%; mixed 8%; no-FDO group: spastic 100%; mixed 0% | FDO group: I 32%; II 36%; III 24%; IV 8%; no-FDO group: I: 36%; II 64% | NR | prognostic |
| Chin et al.^21^ | cross-sectional | USA | NR | Clinic database at the Kennedy Krieger Institute Phelps Centre for CP and Developmental Medicine; included aged ≥18 yr, able to self-report outcome measures, people with mild or severe impairment, CFCS level I-III. | 17 | 31 | 47% | NR | Median (IQR) 4 (2); min-max 1-5 | 24% | prognostic |
| Jensen et al.^57^ | cohort | USA | NR | Recruited consecutively from participants in a previous study on CP and pain; included aged ≥18 yr, reported chronic pain for >3 months, absence of documented severe cognitive impairment. | 50 | 39.6 [18-76] | 50% | spastic: 58%; athetoid 14%; hypotonic 2%; mixed 26% | NR | NR | prognostic |
| Maanum et al.^52^ | cross-sectional | Norway | 2007 to 2008 | Advertisements in newspapers and on the websites of the Norwegian CPs Association and Sunnaas Rehabilitation Hospital; included aged 18-65 yr, unilateral or bilateral CP, GMFCS levels I-III, able to walk independently continuously for 6 minutes, reported declined walking ability compared with adolescence such as  reduced walking distance, increasing stiffness, spasticity, pain,  or balance difficulties. Excluded those with other conditions that could affect the walking ability, orthopaedic surgery within the past 18 months, injections of botulinum  toxin A within the preceding 3 months, pregnancy, ID documented in medical records. | 126 | 39 (12) [18-65] | 58% | spastic unilateral 47%; spastic bilateral 53% | I 10%; II 74%; III 16% | NR | prognostic |
| Malone and Vogtle^49^ | cohort | USA | NR | Adult Day Program of United Cerebral Palsy of Greater Birmingham and persons living independently in the local community (Birmingham, AL); included those who had chronic pain, were self-advocating, and able to respond to questions regarding their health status. | 26 | 42.3 [23-63] | 62% | NR | wheelchair users 54% | NR | prognostic |
| Yamashita et al.^45^ | cross-sectional | Australia | NR | Orthopedics outpatient clinic, a child development support center, and a welfare service facility; included ≥18 yr, cognitive level sufficient to complete the interview and questionnaires. Excluded those with any surgery or botulinum toxin injections in the neck, waist, and upper or lower extremities within 6 months before testing. | 30 | With low back pain 40.1 (14.5); without low back pain 43.7 (17.0) | With low back pain 15%; without low back pain 60% | NR | NR | NR | prognostic |
| Benromano et al.^19^ | cross-sectional | Israel | NR | Individuals with ID recruited from a day care centre for people with ID. Individuals without ID recruited from independent residential communities; individuals with CP with mild-moderate ID and without ID | 18 | With ID: 34.5 (4.9); without ID: 24 (4.2) | 44% | 11% hemiplegia; 17% diplegia; 72% quadriplegia | NR | NR | psychometric |
| Boldingh et al.^58^ | cross-sectional | Netherlands | NR | Nursing homes for severely disabled persons and rehabilitation centres in the Netherlands; included those able to walk independently and with a mental age of ≥4 and were able to use a Faces Pain Scale, assessed using the Columbia Mental Maturity Scale (a nonverbal mental development test). | 160 | 36 [16-84] | 46% | NR | NR | NR | psychometric |
| Jensen et al.^57^ | cross-sectional | USA | NR | Recruited from two studies, a single-assessment survey an a 2-year longitudinal study; included aged ≥18 yr, at most mild cognitive impairment, presence of chronic pain. | 69 | 40.6 (13.1) | 46% | Spastic 58%; athetoid 13%; hypotonic 3%; ataxic 1%; mixed 25%s | ambulatory 17%; Wheelchair user 62%; scooter user 7%; crutches 7%; other 6% | NR | psychometric |
| Tyler et al.^59^ | cross-sectional | USA | NR | Data from two studies associated with an ongoing study of chronic pain management in persons with CP, a 1-time survey and a longitudinal study, were used; included those with IQ of at least 70 or clinical judgement of healthcare provider of no more than mild cognitive impairment. | 50 | 39.7 (13.0) [18-76] | 50% | Spastic 58%; mixed 26%; athetoid 14%; hypotonic 2% | non-ambulatory 80% | NR | psychometric |
| Jacobson et al.^60^ | RCT | Sweden | NR | Tertiary referral center in Stockholm that receives referrals from clinicians at all care levels in Stockholm and adjacent counties, public advertisements in newspapers, patient organisation websites and medical facilities; included aged ≥18 yr, spastic CP, chronic pain related to spastic muscle (duration ≥3 months, intensity ≥3 on Numerical Rating Scale). Excluded those with hypersensitivity to BoNT-A, pregnancy, breastfeeding, treatment with BoNT-A within the last 5 months, changes in muscle-tone–altering medications within the last 2 weeks, clearly degenerative pain mechanisms, and/or ID or communication impairments that disabled the individual from independently giving informed consent | 16 | Intervention group: 24^a^ [18-60]; control group: 33^a^ [21-50] | 63% | Spastic bilateral 69%; unilateral spastic 31% | I-II 56%; III-IV 44% | NR | effectiveness |
| Riquelme et al.^63^ | RCT | Spain | 2010-2011 | Occupational centers established in Majorca and Albacete; included aged 18-40 yr, absence of chronic pain (defined as persistent and generalised pain for >6 months), cognitive level that allow understanding and participating in therapy. | 40 | 30 (4) [22-40] | 35% | ataxic: 22%; bilateral spastic 65%; dystonic 13% | I 30%; II 22%; III 3%; IV 35%; V 11% | 54% | effectiveness |
| Slaman et al.^62^ | RCT | Netherlands | 2009-2011 | Four rehabilitation centers and two rehabilitation departments at university hospitals throughout the western and central regions of the Netherlands; included aged 16-24 yr, spastic CP, GMFCS level I-IV. Excluded those with disabilities other than CP that affect daily physical activity or cardiopulmo- nary fitness; contraindication to (maximal) exercise; physical activity level at baseline exceeds the mean physical activity level +2 SD of a CP population corresponding with 263 minutes of physical activity per day; severe cognitive disorder or insufficient comprehension of Dutch Language that would impede understanding of instructions. | 57 | 20 (3) | 53% | spastic 100% | I 58%; II 32%; III 9%; IV 2% | NR | effectiveness |
| Vidailhet et al.^64^ | pre- post- intervention | France | 2003-2006 | Multicentre; included those with disabling dystonia, defined as involuntary sustained muscle contractions that led to abnormal movements and postures, which could be multifocal or generalised, with a combination of segmental crural dystonia (one leg and the trunk) and involvement of any other segment (face, neck, or upper or lower limbs); no psychiatric disorders, little or no cognitive impairment (mini-mental state examination [MMSE] score >24; neonatal hypoxic or ischaemic encephalopathy and delayed early motor milestones; no other cause of dystonia; little or no spasticity (Ashworth score <2 for each segment); no more than slight abnormalities seen on T1-weighted MRI (decreased grey–white matter contrast with partial disappearance of the basal ganglia and minimum atrophy of the pallidum or putamen); optimum pharmacological treatments (i.e., the highest tolerated doses of drugs known to be useful in dystonia, including levodopa and anticholinergics) were ineffective | 13 | 33^a^ [20-44] | 10% | generalised dystonia 100% | NR | NR | effectiveness |
| Yi et al.^61^ | RCT | Korea | NR | Included aged >20 yr, with dyskinesia, with cervical dystonia. | 17 | 46.0 (6.4) | 50% | dyskinesia 100% | I 25%; II 44%; III 0%; IV 25%; V 6.2% | NR | effectiveness |

Note: Although extracted, we do not report the following participant characteristics in the table because less than 6 studies provided this information: MACS level, CFCS level, EDACS level, socioeconomic status, ethnicity, BMI.

SD: Standard deviation; GMFCS: Gross Motor Function Classification System; ID: Intellectual Disability; FDO: femoral derotation osteotomy; NR: not reported; SDR: selective dorsal rhizotomy; CFCS: Communication Function Classification System; RCT: randomised controlled trial

^a^Median (interquartile range)

^b^Mobility status reported if GMFCS level not reported

^c^Objective(s) that the study is addressing: prevalence=objective 1, prognostic=objective 2, psychometric= objective 3, effectiveness=objective 4

^d^participant characteristics only reported for those with chronic pain (n=149)

**REFERENCES**

78. Benner JL, Hilberink SR, Veenis T, Stam HJ, van der Slot WM, Roebroeck ME. Long-Term Deterioration of Perceived Health and Functioning in Adults With Cerebral Palsy. Arch Phys Med Rehabil. 2017;98(11):2196-205.

79. Dauvergne F, Eon Y, Gallien P, Bouric S, Duruflé-Tapin A, Cambla N, Nicolas B. Disabilities, access to medical care, and way of life of adults with cerebral palsy. APIB study: first results. Ann Readapt Med Phys. 2007;50(1):20-27. doi:10.1016/j.annrmp.2006.06.008.

80. Botura C de A, Ames FQ, Botura AC de A, Bersani-Amado LE, Bardini AVSL, Cuman RK. Pain symptoms in patients with severe cerebral palsy: Prevalence among patients with higher degree of locomotor impairment. Trop J Pharm Res 2017;16(6):1431-6.

81. García Jalón EG, Maguire A, Perra O, Gavin A, O'Reilly D, Thurston A. Data linkage and pain medication in people with cerebral palsy: A cross-sectional study. Dev Med Child Neurol 2021;63(9):1085-92.

82. Marco Götze, MD, Andreas Geisbüsch, MD, Mirjam Thielen, MD, Leonhard Döderlein, MD, Sebastian I. Wolf, Thomas Dreher, MD, and Cornelia Putz, MD. Pain in Adults With Cerebral Palsy After Single-Event Multilevel Surgery.Am J Phys Med Rehabil 2022;101:119–123.

84. Thorpe D, Gannotti M, Peterson MD, Wang C-H, Freburger J. Musculoskeletal diagnoses, comorbidities, and physical and occupational therapy use among older adults with and without cerebral palsy. Disabil Health J 2021;14(2):101109.
