# Supplementary material for: Pain in adults with cerebral palsy: A systematic review
Source: Dev Med Child Neurol. 2025 Feb 12;67(7):854–74. doi: 10.1111/dmcn.16254 (PMC12134420; doi:10.1111/dmcn.16254)
Supplement: Supplementary file 5 — Table S2: Quality appraisal of prevalence studies reporting prevalence of pain. [file DMCN-67-854-s005.docx]

Supplemental table 2 Quality appraisal of prevalence studies reporting prevalence of pain

| Study | Was the sample frame appropriate to address the target population? | Were study participants sampled in an appropriate way? | Was the sample size adequate? | Were the study subjects and the setting described in detail? | Was the data analysis conducted with sufficient coverage of the identified sample? | Were valid methods used for the identification of the condition? | Was the condition measured in a standard, reliable way for all participants? | Was there appropriate statistical analysis? | Was the response rate adequate, and if not, was the low response rate managed appropriately? |
| --- | --- | --- | --- | --- | --- | --- | --- | --- | --- |
| Asuman et al.^13^ | yes | yes | yes | no | yes | unclear | unclear | no | yes |
| Benner et al. ^78^ | yes | yes | no | yes | yes | unclear | yes | no | yes |
| Bourelle et al.^20^ | no | unclear | no | no | yes | unclear | unclear | yes | yes |
| Dauvergne et al.^79^ | yes | yes | yes | no | unclear | no | unclear | no | no |
| de Albuquerque et al.^80^ | no | yes | no | no | yes | unclear | unclear | yes | unclear |
| du Toit et al.^26^ | no | no | no | no | yes | unclear | yes | no | yes |
| Eken et al.^27^ | no | yes | no | no | yes | unclear | unclear | no | unclear |
| Engel et al.^77^ | no | yes | no | yes | yes | unclear | unclear | yes | unclear |
| Flanigan et al.^53^ | yes | yes | no | no | no | unclear | yes | no | unclear |
| Frank and De Souza^21^ | no | no | no | no | unclear | no | unclear | yes | unclear |
| Gallien et al.^22^ | unclear | yes | yes | no | unclear | unclear | yes | yes | yes |
| Garca Jalon et al.^81^ | yes | yes | yes | yes | yes | yes | yes | no | yes |
| Gotze et al.^82^ | no | yes | no | no | no | yes | yes | yes | no |
| Hilberink et al.^75^ | no | yes | no | yes | yes | unclear | yes | no | yes |
| Hirsh et al.^71^ | no | no | no | yes | unclear | unclear | unclear | no | no |
| Hung et al.^73^ | yes | yes | yes | no | yes | no | yes | no | unclear |
| İçağasıoğlu et al.^43^ | yes | yes | no | yes | yes | unclear | unclear | yes | unclear |
| Jacobson et al.^35^ | no | yes | no | no | yes | yes | yes | no | yes |
| Jahnsen e al.^32^ | no | yes | yes | yes | yes | unclear | yes | yes | no |
| Jarl et al.^4^ | yes | yes | yes | no | yes | unclear | yes | yes | yes |
| Jonsson et al.^34^ | yes | unclear | no | yes | unclear | unclear | yes | yes | unclear |
| Langerak et al.24 | no | yes | no | no | yes | unclear | yes | no | yes |
| Lundkvist Josenby and Westbom^56^ | no | yes | no | yes | yes | unclear | yes | no | unclear |
| Murphy et al.^72^ | yes | no | no | no | yes | unclear | yes | yes | unclear |
| Noonan et al.^54^ | no | unclear | no | no | yes | unclear | yes | no | yes |
| Noonan et al.^54^ | no | unclear | no | no | yes | unclear | yes | no | yes |
| Opheim et al.^31^ | no | unclear | no | yes | unclear | unclear | yes | yes | yes |
| Opheim et al.^44^ | no | unclear | no | yes | yes | unclear | yes | yes | yes |
| Park and Kim^46^ | unclear | yes | no | no | no | unclear | unclear | no | no |
| Patatoukas et al.^76^ | no | yes | no | no | unclear | unclear | unclear | yes | yes |
| Peterson et al.^38^ | unclear | yes | no | no | no | yes | yes | yes | unclear |
| Peterson et al.^39^ | yes | yes | yes | no | yes | unclear | yes | yes | yes |
| Rodby-Bousquet et al.^42^ | yes | yes | no | no | unclear | unclear | yes | yes | yes |
| Rodby-Bousquet et al.10 | yes | yes | yes | yes | yes | unclear | yes | yes | yes |
| Salie et al.^74^ | no | no | no | no | unclear | yes | yes | no | unclear |
| Sandstrom et al.^47^ | yes | yes | no | no | yes | unclear | yes | yes | no |
| Sarmiento et al.^30^ | unclear | yes | no | no | yes | unclear | yes | no | yes |
| Schmidt et al.^37^ | no | yes | no | no | yes | unclear | yes | unclear | unclear |
| Shrader et al.^33^ | no | yes | no | no | yes | yes | yes | no | no |
| Sienko^48^ | yes | yes | no | no | yes | unclear | yes | no | no |
| Tedroff et al.^28^ | no | yes | no | no | yes | unclear | Unclear | yes | yes |
| Terjesen et al.^51^ | yes | yes | no | no | yes | unclear | unclear | unclear | no |
| Thorpe et al.^84^ | no | yes | yes | no | yes | yes | yes | yes | yes |
| Turk et al.^50^ | no | yes | no | yes | yes | unclear | yes | yes | unclear |
| Van Der Slot et al.^29^ | no | yes | no | yes | yes | unclear | yes | yes | no |
| van Gorp et al.^40^ | no | unclear | no | yes | yes | yes | yes | no | no |
| Veerbeek et al.^25^ | no | yes | no | no | yes | unclear | yes | no | yes |
| Vidart et al.^6^ | yes | yes | no | yes | yes | unclear | yes | no | yes |
| Whitney et al.^12^ | yes | yes | yes | no | yes | unclear | yes | no | unclear |

**REFERENCES**

78. Benner JL, Hilberink SR, Veenis T, Stam HJ, van der Slot WM, Roebroeck ME. Long-Term Deterioration of Perceived Health and Functioning in Adults With Cerebral Palsy. Arch Phys Med Rehabil. 2017;98(11):2196-205.

79. Dauvergne F, Eon Y, Gallien P, Bouric S, Duruflé-Tapin A, Cambla N, Nicolas B. Disabilities, access to medical care, and way of life of adults with cerebral palsy. APIB study: first results. Ann Readapt Med Phys. 2007;50(1):20-27. doi:10.1016/j.annrmp.2006.06.008.

80. Botura C de A, Ames FQ, Botura AC de A, Bersani-Amado LE, Bardini AVSL, Cuman RK. Pain symptoms in patients with severe cerebral palsy: Prevalence among patients with higher degree of locomotor impairment. Trop J Pharm Res 2017;16(6):1431-6.

81. García Jalón EG, Maguire A, Perra O, Gavin A, O'Reilly D, Thurston A. Data linkage and pain medication in people with cerebral palsy: A cross-sectional study. Dev Med Child Neurol 2021;63(9):1085-92.

82. Marco Götze, MD, Andreas Geisbüsch, MD, Mirjam Thielen, MD, Leonhard Döderlein, MD, Sebastian I. Wolf, Thomas Dreher, MD, and Cornelia Putz, MD. Pain in Adults With Cerebral Palsy After Single-Event Multilevel Surgery.Am J Phys Med Rehabil 2022;101:119–123.

84. Thorpe D, Gannotti M, Peterson MD, Wang C-H, Freburger J. Musculoskeletal diagnoses, comorbidities, and physical and occupational therapy use among older adults with and without cerebral palsy. Disabil Health J 2021;14(2):101109.
