# Supplementary material for: Pain in adults with cerebral palsy: A systematic review
Source: Dev Med Child Neurol. 2025 Feb 12;67(7):854–74. doi: 10.1111/dmcn.16254 (PMC12134420; doi:10.1111/dmcn.16254)
Supplement: Supplementary file 6 — Table S3: Quality appraisal of cross‐sectional studies comparing pain prevalence between adults with and without cerebral palsy. [file DMCN-67-854-s014.docx]

Supplemental table 3 Quality appraisal of cross sectional studies comparing pain prevalence between adults with and without cerebral palsy

| Study | Were the criteria for inclusion in the sample clearly defined? | Were the study subjects and the setting described in detail? | Were objective, standard criteria used for measurement of the condition? | Were confounding factors identified? | Were strategies to deal with confounding factors stated? | Were the outcomes measured in a valid and reliable way? | Was appropriate statistical analysis used? |
| --- | --- | --- | --- | --- | --- | --- | --- |
| duToit et al.^26^ | yes | no | unclear | yes | yes | unclear | no |
| Jahnsen et al.^32^ | no | yes | unclear | yes | yes | unclear | no |
| Opheim et al.^31^ | no | yes | yes | no | no | unclear | yes |
| Peterson et al.^39^ | yes | no | unclear | yes |  | yes | yes |
| van der slot et al.^29^ | no | yes | unclear | no | no | unclear | no |
| van Gorp et al.^40^ | yes | yes | unclear | yes | yes | yes | yes |
