# Supplementary material for: Pain in adults with cerebral palsy: A systematic review
Source: Dev Med Child Neurol. 2025 Feb 12;67(7):854–74. doi: 10.1111/dmcn.16254 (PMC12134420; doi:10.1111/dmcn.16254)
Supplement: Supplementary file 7 — Table S4: Quality appraisal of cross‐sectional studies examining prognostic factors for pain. [file DMCN-67-854-s006.docx]

Supplemental table 4 Quality appraisal of cross sectional studies examining prognostic factors for pain

| Study | Were the criteria for inclusion in the sample clearly defined? | Were the study subjects and the setting described in detail? | Was the exposure measured in a valid and reliable way? | Were objective, standard criteria used for measurement of the condition? | Were confounding factors identified? | Were strategies to deal with confounding factors stated? | Were the outcomes measured in a valid and reliable way? | Was appropriate statistical analysis used? |
| --- | --- | --- | --- | --- | --- | --- | --- | --- |
| Chin et al.^41^ | yes | no | yes | unclear | no | no | unclear | yes |
| Eken et al.^27^ | yes | no | yes | unclear | no | no | unclear | yes |
| Flanigan et al.^53^ | yes | yes | yes | yes | yes | yes | unclear | yes |
| İçağasıoğlu et al.^43^ | unclear | no | yes | unclear | no | no | no | yes |
| Jacobson et al.^35^ | unclear | no | yes | yes | no | no | unclear | yes |
| Jahnsen et al.^32^ | yes | no | yes | unclear | no | no | unclear | yes |
| Jarl et al.^36^ | unclear | no | yes | unclear | no | no | no | yes |
| Jonsson et al.^34^ | yes | yes | yes | unclear | no | no | unclear | yes |
| Maanum et al.^52^ | yes | yes | yes | unclear | yes | yes | yes | yes |
| Noonan et al.^54^ | no | no | yes | unclear | no | no | unclear | yes |
| Opheim et al.^44^ | yes | yes | yes | yes | no | no | unclear | yes |
| Park and Kim^46^ | yes | no | unclear | unclear | no | no | unclear | yes |
| Rodby-Bousquet et al.^42^ | unclear | no | yes | yes | no | no | unclear | yes |
| Rodby-Bousquet et al.^10^ | yes | no | yes | unclear | yes | yes | unclear | yes |
| Sandstrom et al.^47^ | unclear | no | yes | unclear | no | no | unclear | yes |
| Sarmiento et al.^30^ | yes | no | yes | unclear | no | no | unclear | yes |
| Sienko^48^ | yes | no | yes | unclear | no | no | unclear | yes |
| Terjesen et al.^51^ | yes | no | yes | unclear | no | no | unclear | yes |
| Turk et al.^50^ | no | yes | yes | yes | no | no | unclear | yes |
| van der Slot et al.^29^ | yes | yes | yes | unclear | yes | yes | unclear | yes |
| Van Gorp et al.^40^ | unclear | yes | yes | unclear | no | no | yes | yes |
| Yamashita et al.^45^ | yes | no | unclear | unclear | no | no | unclear | yes |
