# Supplementary material for: Pain in adults with cerebral palsy: A systematic review
Source: Dev Med Child Neurol. 2025 Feb 12;67(7):854–74. doi: 10.1111/dmcn.16254 (PMC12134420; doi:10.1111/dmcn.16254)
Supplement: Supplementary file 8 — Table S5: Quality appraisal of cohort studies examining prognostic factors for pain. [file DMCN-67-854-s016.docx]

Supplemental table 5 Quality appraisal of cohort studies examining prognostic factors for pain

| Study | Were the two groups similar and recruited from the same population? | Were the exposures measured similarly to assign people to both exposed and unexposed groups? | Were confounding factors identified? | Were strategies to deal with confounding factors stated? | Were the groups/participants free of the outcome at the start of the study (or at the moment of exposure)? | Were the outcomes measured in a valid and reliable way? | Was the follow up time reported and sufficient to be long enough for outcomes to occur? | Was follow up complete, and if not, were the reasons to loss to follow up described and explored? | Were strategies to address incomplete follow up utilized? | Was appropriate statistical analysis used? |
| --- | --- | --- | --- | --- | --- | --- | --- | --- | --- | --- |
| Boyer et al.^55^ | yes | yes | yes | yes | unclear | unclear | yes | no | no | yes |
| duToit et al.^26^ | yes | yes | no | no | no | unclear | unclear | yes | no | no |
| Jensen et al.^57^ | yes | yes | no | no | no | unclear | yes | yes | no | no |
| Lundkvist and Westbom^56^ | yes | yes | no | no | yes | unclear | yes | yes | no | yes |
| Malone and Vogtle^49^ | yes | yes | no | no | no | unclear | no | yes | no | yes |
| Opheim et al.^31^ | yes | yes | no | no | yes | unclear | yes | no | no | yes |
