# Supplementary material for: Pain in adults with cerebral palsy: A systematic review
Source: Dev Med Child Neurol. 2025 Feb 12;67(7):854–74. doi: 10.1111/dmcn.16254 (PMC12134420; doi:10.1111/dmcn.16254)
Supplement: Supplementary file 9 — Table S6: Quality appraisal of studies examining psychometric properties of pain assessment tools. [file DMCN-67-854-s013.docx]

Supplemental table 6 Quality appraisal of studies examining psychometric properties of pain assessment tools

| Study | Was an internal consistency statistic calculated for each unidimensional scale or subscale separately | For continuous scores: Was Cronbach’s alpha or omega calculated? | For dichotomous scores: Was Cronbach’s alpha or KR‐ 20 calculated? | For IRT‐based scores: Was standard error of the theta (SE (θ)) or reliability coefficient of estimated latent trait value (index of (subject or item) separation) calculated? | Were there any other important flaws in the design or statistical methods of the study? | Is it clear what the comparator instrument(s) measure(s)? | Were the measurement properties of the comparator instrument(s) sufficient? | Was the statistical method appropriate for the hypotheses to be tested | Were there any other important flaws in the design or statistical methods of the study? |
| --- | --- | --- | --- | --- | --- | --- | --- | --- | --- |
| Benromano et al.^19^ | NA | NA | NA | NA | NA | adequate | adequate | adequate | doubtful |
| Boldingh et al.^58^ | adequate | adequate | NA | NA | inadequate | adequate | inadequate | adequate | inadequate |
| Jensen et al.^57^ | NA | NA | NA | NA | NA | adequate | inadequate | adequate | inadequate |
| Tyler et al.^59^ | adequate | adequate | NA | NA | inadequate | adequate | inadequate | doubtful | doubtful |
