# Supplementary material for: Pain in adults with cerebral palsy: A systematic review
Source: Dev Med Child Neurol. 2025 Feb 12;67(7):854–74. doi: 10.1111/dmcn.16254 (PMC12134420; doi:10.1111/dmcn.16254)
Supplement: Supplementary file 10 — Table S7: Quality appraisal of randomized controlled trials examining effectiveness of interventions. [file DMCN-67-854-s020.docx]

Supplemental table 7 Quality appraisal of randomised controlled trials examining effectiveness of interventions

| study | Was true randomization used for assignment of participants to treatment groups? | Was allocation to treatment groups concealed? | Were treatment groups similar at the baseline? | Were participants blind to treatment assignment? | Were those delivering the treatment blind to treatment assignment? | Were treatment groups treated identically other than the intervention of interest? | Were outcome assessors blind to treatment assignment? | Were outcomes measured in the same way for treatment groups? | Were outcomes measured in a reliable way? | Was follow up complete and if not, were differences between groups in terms of their follow up adequately described and analysed? | Were participants analysed in the groups to which they were randomized? | Was appropriate statistical analysis used? | Was the trial design appropriate and any deviations from the standard RCT design (individual randomization, parallel groups) accounted for in the conduct and analysis of the trial? |
| --- | --- | --- | --- | --- | --- | --- | --- | --- | --- | --- | --- | --- | --- |
| Jacobson et al.^60^ | yes | no | no | yes | yes | yes | yes | yes | unclear | yes | yes | no | yes |
| Riquelme et al.^63^ | unclear | unclear | unclear | no | no | yes | yes | yes | yes | no | unclear | no | yes |
| Slaman et al.^62^ | yes | unclear | yes | no | no | unclear | yes | yes | yes | yes | unclear | yes | yes |
| Yi et al.^61^ | unclear | unclear | unclear | unclear | unclear | unclear | unclear | unclear | unclear | no | no | no | no |
