# Supplementary material for: Pain in adults with cerebral palsy: A systematic review
Source: Dev Med Child Neurol. 2025 Feb 12;67(7):854–74. doi: 10.1111/dmcn.16254 (PMC12134420; doi:10.1111/dmcn.16254)
Supplement: Supplementary file 11 — Table S8: Quality appraisal of quasi‐experimental studies examining effectiveness of interventions. [file DMCN-67-854-s004.docx]

Supplemental table 8 Quality appraisal of quasi-experimental studies examining effectiveness of interventions

| Study | Is it clear in the study what is the ‘cause’ and what is the ‘effect’ (i.e. there is no confusion about which variable comes first)? | Were the participants included in any comparisons similar? | Were the participants included in any comparisons receiving similar treatment/care, other than the exposure or intervention of interest? | Was there a control group? | Were there multiple measurements of the outcome both pre and post the intervention/exposure? | Was follow up complete and if not, were differences between groups in terms of their follow up adequately described and analysed? | Were the outcomes of participants included in any comparisons measured in the same way? | Were outcomes measured in a reliable way? | Was appropriate statistical analysis used? |
| --- | --- | --- | --- | --- | --- | --- | --- | --- | --- |
| Vidailhet et al.^64^ | yes | NA | NA | no | no | NA | yes | unclear | yes |
