# Supplementary material for: Pain in adults with cerebral palsy: A systematic review
Source: Dev Med Child Neurol. 2025 Feb 12;67(7):854–74. doi: 10.1111/dmcn.16254 (PMC12134420; doi:10.1111/dmcn.16254)
Supplement: Supplementary file 12 — Table S9: Summary of findings: prevalence of pain among adults with cerebral palsy. [file DMCN-67-854-s015.docx]

**TABLE S9** Summary of findings: prevalence of pain among adults with cerebral palsy

| **Study** | **Pain assessment and timeframe** | **Respondent** | **Sample size** | **Country** | **Context** | **Pain, *n* (%)** | **Pain location, *n* (%)^a^** | **Pain frequency^a^** | **Pain duration^a^** |
| --- | --- | --- | --- | --- | --- | --- | --- | --- | --- |
| Asuman et al.^13^ | Pain in a specific year was defined as having a pain diagnosis in the National Patient Register (in- and outpatient care) or being dispensed pain-related prescription medication as registered in the Pharmaceutical Register. Definition of pain in this study includes medication and diagnoses of both CP- and non-CP-related pain. Over-the-counter pain medications without prescription were not included. | NR | 6899 | Sweden | Population-based | 38% | NR | NR | NR |
| Benner et al.^78^ | McGill Pain Questionnaire assessed presence of pain and pain intensity. Presence of pain was scored either yes or no; least and worst pain intensity were scored on a horizontal 100-mm VAS. | Self-report 61%; assisted self-report 21%; proxy-report 18% | 49 | The Netherlands | Clinic-based | 71% | NR | NR | NR |
| Bourelle et al.^20^ | NR | NR | 17 | France | Clinic-based | 4 (24%) | NR | NR | NR |
| Dauvergne et al.^79^ | Survey questionnaire. | NR | 562 | France | Population-based | 75% | NR | Sometimes 40%; often 25%; always 10% | NR |
| de Albuquerque et al.^80^ | Inventário de Comportamentos da Dor na Deficiência Neurológica (Portugeuse version of Paediatric Pain Profile) consisting of 20 items that describe pain-related behaviours in patients; ‘current pain’. | Proxy-report | 50 | Brazil | Population-based | 58% | NR | NR | NR |
| du Toit et al.^26^ | Frequency of pain was assessed on the basis of a pain frequency questionnaire that asked participants to indicate how often they experience pain in their back (cervical, thoracic, and lumbosacral regions), their upper limbs (shoulder and arm), and their lower limbs (leg, hip, and knee). Responses were categorized as ‘never’, ‘occasionally’, ‘weekly’, and ‘daily’. The highest pain frequency was recorded for each level, i.e. back, upper, and lower limb. | Self-report | 28 | South Africa | Clinic-based | NR | Back 75% | Daily back pain 18%; weekly back pain 5% | NR |
| Eken et al.^27^ | Frequency of pain was captured by asking participants to indicate how often they experienced pain in their back (head/neck, thoracic and lumbar spine level), upper limbs (shoulder, arm) and lower limbs (leg, hip, knee). The frequency was divided into ‘never’, ‘occasionally’, ‘weekly’, and ‘daily’. | Self-report | 30 | South Africa | Clinic-based | NR | Spine 83%; upper limb 27%; lower limb 57% | Spine: 20% daily, 13% weekly, 50% occasionally; upper limb 10% weekly, 17% occasionally; lower limb: 24% daily, 3% weekly, 30% occasionally | NR |
| Engel et al.^77^ | Asked to indicate whether they experienced a pain problem during the previous 3 months. | Self-report | 100 | USA | Clinic-based | 67 (67%) | Low back 47%; hip 41%; leg 35%; hand and wrist 35%; neck 33%; shoulder 30%; arm 27%; upper back 23%; feet/ankle 26%; head 21%; abdominal/pelvis 14%; buttocks 13%; chest 11% | 24% ‘constant’ pain; 19% daily pain but some pain-free periods | Low back mean (SD) 13.1 months (10.8) ; hip 17.1 months (15.1); leg 16.9 months (12.6) |
| Flanigan et al.^53^ | Brief Pain Inventory; pain in previous week. | Self-report 85%; proxy-report 15% | 47 | USA | Clinic-based | 26 (55%) | NR | NR | NR |
| Frank et al.^21^ | Users with pain requiring further investigation/management or influencing electric-powered chair prescription were recorded as problematic pain. | NR | 79 | UK | Clinic-based | 19 (24%) | NR | NR | NR |
| Gallien et al.^22^ | NR | NR | 476 | France | NR | 424 (89%) | NR | Sometimes 48%; often 29%; always 12% | NR |
| Garca Jalon et al.^81^ | Pain medication used as a proxy for experiencing pain. Focus on pain specific medication: non-steroidal anti-inflammatories, opioid analgesics, and non-opioid analgesics. | NR | 742 | Northern Ireland | Population-based | 285 (38%) | NR | NR | NR |
| Gotze et al.^82^ | Questionnaire included the current VAS, need for pain medication, origin and duration of pain; number reporting ‘persistent pain’. | NR | 24 | Germany | Clinic-based | 16 (67%) | Foot 25%; knee 25%; spine 17%; hip 13%; shoulder/arm 13%; iliosacral joint 8%; leg 8% | NR | <1 month 25%; 6–12 months 12.5%; >12 months 29.2% |
| Hilberink et al.^75^ | Pain severity and impact of pain were assessed with two sections of the Dutch version of the McGill Pain Questionnaire. Adults were asked to indicate the pain severity when they suffered pain the least, the most, and currently on a VAS, with illustrations of a neutral and painful face at either end of a 100-mm long line. | Self-report 74%; proxy-report 26% | 54 | The Netherlands | Clinic-based | 32 (59%) | Hip or leg 19%; back 17%; neck, shoulder, and arm 15% | NR | NR |
| Hirsh et al.^71^ | Survey or interview asking whether they currently experienced pain or experienced pain in previous 3 months; average pain intensity over previous week (0 [no pain] to 10 [pain as bad as could be]); bodily locations where they experienced persistent ‘bothersome’ pain. | Self-report | 83 | USA | Population-based | 52 (63%) | Lower back 45%; legs 36%; hips 36%; feet 34%; neck 31%; shoulder 50%; knees 31%; hands 27%; upper back 25%; wrists 20%; buttocks 20%; ankles 19%; elbows 16%; head 13%; abdomen/pelvis 12%; chest 5% | NR | NR |
| Hung et al.73 | NR | NR | 32 | USA | Clinic-based | NR | Neck 50% |  | NR |
| İçağasıoğlu et al.^43^ | Patient data were collected from the patients themselves and/or from their family members for those with speech disorder. | Self- or proxy-report | 70 | Turkey | Clinic-based | 22 (31%) | NR | NR | NR |
| Jacobson et al.^35^ | SF-36 version 2; ‘mild bodily pain’ in previous 4 weeks. | Self-report 61%; proxy-report 39% | 61 | Sweden | Population-based | (49%) | NR | NR | NR |
| Jahnsen et al.^32^ | SF-36, bodily pain domain. Asked items on pain localization, frequency, and duration. Chronic pain defined as daily pain for 1 year or more. | NR | 398 | Norway | Population-based | 327 (82%) | Back 59%; neck 44%; foot/ankle 44%; shoulder 43%; knee 39%; hip 36%; arm 28%; head 26%; other 6% | NR | Daily pain for ≥1 year 28% |
| Jarl et al.^4^ | NR | Self-report | 408 | Sweden | Population-based | 273 (67%) | NR | NR | NR |
| Jonsson et al.^34^ | Semi-structured interview; current or recurring pain of any type at the time of assessment Participants were asked to describe pain intensity, (mild, moderate, or severe), frequency (daily, weekly, monthly, or more seldom), and duration (more or less than 3 months). When participants reported several different pain sites, with different frequency, intensity, or duration, the highest intensity and frequency, and the longest duration, were recorded. | Self-report | 153 | Sweden | Population-based | 100 (65%) | NR | monthly 8%; weekly 15%; daily 31%; unknown 11% | <3 months 5%; >3 months 49%; unknown 11% |
| Langerak et el.^24^ | Self-developed pain questionnaire. | Self-report | 30 | Germany | Clinic-based | NR | Cervical 63%; thoracic 33%; lumbosacral 60%; arm 10%; shoulder 23%; leg 47%; hip 37%; knee 37% | Daily neck pain 0%; daily lower back pain 20%; daily lower extremity pain 13% | NR |
| Lundkvist and Westbom et al.^56^ | Presence of pain was assessed by either the person him/herself or by proxy. For adults (>18 years) recalling the previous 4 weeks for spinal pain that was classified as being moderate or severe. | Self- or proxy-report | 66 | Sweden | Population-based | NR | Spine: 18% at 20 years; 20% at 25 years | NR | NR |
| Murphy et al.^72^ | Interviews and examinations. | Self-report | 101 | USA | Population-based | NR | Cervical 46%; back 36%; weight bearing joints 23% | NR | NR |
| Noonan et al.^54^ | Interview with primary caregiver; asked whether, in his or her opinion, the hips of the subject were painful and frequency of pain. There were six potential answers: (1) the left hip was painful; (2) the right hip was painful; (3) both hips were painful; (4) at least one of the hips was painful but the caregiver was unsure which hip was painful; (5) the caregiver was not sure whether the subject had any pain; and (6) neither hip was painful. | Proxy-report | 77 | USA | Residential centres | NR | Hip 49% | NR | NR |
| Opheim et al.^31^ | Self-reported questionnaire; the number of pain sites was reported with checkboxes for eight body parts, and pain frequency was reported as never, seldom, monthly, weekly, or daily. Daily pain for more than 1 year was considered chronic. | Self-report | 149 | Norway | Population-based | 31% | NR | NR | Daily pain for ≥1 year 24% |
| Opheim et al.^31^ | Self-reported questionnaire; the number of pain sites was reported with checkboxes for eight body parts, and pain frequency was reported as never, seldom, monthly, weekly, or daily. Daily pain for more than 1 year was considered chronic. | Self-report | 149 | Norway | Population-based | 83% | NR | NR | Median (IQR) 16 years (7–25)^d^ |
| Park and Ekim et al.^46^ | ‘General pain’; assessment not reported. | NR | 52 | Korea | Population-based | 20 (38%) | NR | NR | NR |
| Patatoukas et al.^76^ | Questionnaire about sports-related injuries and illnesses experienced in the athlete’s sports life, including site of injury, history, signs, and symptoms of the pain in any body part, shown in a body figure, the mechanisms of injury sustained, the period (training or competition) when the injury occurred. Athletic injuries were defined as soft tissue injuries, fractures, contusions, lacerations, blisters, ruptures, low back pain. | NR | 33 | Greece | NR | NR | Low back 0% | NR | NR |
| Peterson et al.^38^ | Neuropathic pain was assessed using the painDETECT Questionnaire, a 13-item survey to determine the presence/severity of pain of neuropathic origin; the degree of centrally enhanced pain processing was assessed using the American College of Rheumatology 2011 FM Survey criteria including widespread body pain and comorbid somatic symptoms (higher scores indicating greater centralized pain); pain severity was measured with the Brief Pain Inventory, which assessed the overall current pain and average, least, and worst body pain in the previous 24 hours (11-point Likert-type scale, total represents the average score across all responses, and ranges from 0 to 10 with higher scores indicating greater pain severity). | Self-reported | 71 | USA | Clinic-based | 62%; 33.8% nociplastic; 11.3% neuropathic; 15.5% mixed | NR | NR | Nociplastic-type mean 1.9 (2.19); mixed neuropathic/nociplastic-type mean 5.86 (2.44) |
| Peterson et al.^39^ | Used Medical Expenditure Panel Survey, an ongoing nationally representative survey of the US civilian, non-institutionalized population conducted annually by the Agency for Healthcare Research and Quality. Data collected by interviews with a single respondent for the household. | NR | 1015 | USA | Population-based | 43.6% (95% CI 39.4–47.7) (age adjusted) | NR | NR | NR |
| Rodby-Bousquet et al.^42^ | Pain was reported either by client or by proxy as yes or no to any presence of pain during the previous 4 weeks. | Self- or proxy-report | 102 | Sweden | Population-based | 63 (62%) | NR | NR | NR |
| Rodby-Bousquet et al.^10^ | Prevalence of pain (‘Do you experience pain?’) was either self- or proxy-reported as ‘yes’ or ‘no’. If pain was reported, pain severity was rated for the following 10 body sites: neck; back/spine; shoulder; arm/hand; hip/thigh; knee; feet/lower leg; head; stomach; or other location. As applicable, pain severity (‘How much bodily pain have you had during the past 4 weeks?’) was graded according to the SF-36 into one of the following response options for each relevant pain site: 1 = none; 2 = very mild; 3 = mild; 4 = moderate; 5 = severe; 6 = very severe. | Self-report 71%; proxy-report 29% | 1591 | Sweden | Population-based | 1059 (67%) | Back 33%; hips/thighs 28%; feet/lower legs 24%; knees 23%; shoulders 21%; arms/hands 18%; neck 21%; head 20%; stomach 19% | NR | NR |
| Salie et al.^74^ | Questionnaire used to collect data about the prevalence of pain at back (cervical, thoracic, and lumbosacral), upper limb (arm and shoulder), and lower limb (leg, hip, knee, and ankle) levels. At each site the highest pain frequency that was reported was categorized into never, occasionally, weekly, or daily. | NR | 30 | South Africa | Population-based | NR | Back 90%; upper limb 37%; lower limb 70% | NR | NR |
| Sandstrom et al.^47^ | Pain localization was described by means of given alternatives (neck, upper extremity, back, lower extremity). Pain intensity was rated on a VAS from 0mm (no pain) to 100mm (worst imaginable pain). The individuals rated pain intensity as it was when it was at its worst and at its best. | Self-report 81%; proxy-report 19% | 48 | Sweden | Clinic-based | 33 (69%) | Neck 21%; upper extremity 25%; back 35%; lower extremity 46% | NR | NR |
| Sarmiento et al.^30^ | Chronic pain defined as pain that is persistent for more than 3 months; those who reported chronic pain were asked questions on pain location, Brief Pain Inventory (pain intensity categorized as mild [1–4], moderate [5–6], and severe [7–10]), PROMIS Pain interference survey. | Self-report | 263 | USA | Population-based | 205 (78%) | Head 23%; face 8%; neck 52%; shoulder 50%; arm 34%; upper back 28%; chest 9%; abdomen 16%; lower back 76%; hand 41%; buttock 22%; hip 65%; knee 57%; leg 60%; foot 46% | NR | NR |
| Schmidt et al.^37^ | NR | Self-report 43%; assisted self-report  27%; proxy-report  30% | 198 | France and Germany | Population-based | 146 (74%) | NR | NR | NR |
| Shrader et al.^33^ | Survey question; ‘chronic pain’. | Self-report 67%; proxy-report 33% | 126 | USA | Clinic-based | 62 (49%) | Joint/muscle 68% | NR | NR |
| Sienko^83^ | The magnitude of pain was assessed with the pain question from the 2011 Behavioral Risk Factor Surveillance System. The System’s pain question assesses pain on a 5-point scale, with higher scores indicating more pain. | Self-report 59%; proxy-report 41% | 97 | USA | Clinic-based | 85% | NR | NR | NR |
| Tedroff et al.^28^ | Swedish version of Brief Pain Inventory; it comprises two parts. The first part contains eight items about pain location, pain severity, analgesics used, and pain relief. In the second part, the individual is asked about pain interference with activities of daily life (seven items). A composite score is obtained for pain severity and pain interference, where 0 implies the least and 10 the worst situation. | NR | 18 | Sweden | Clinic-based | 9 (50%) | NR | NR | NR |
| Terjesen et al.^51^ | ‘Mild to moderate pain lasting several months in the back, hips or further distally’; assessment not reported. | NR | 37 | Norway | Clinic-based | 15 (41%) | NR | NR | NR |
| Thorpe et al.^84^ | Pain identified using the International Classification of Diseases Ninth Revision, Clinical Modification diagnosis codes associated with all ambulatory claims using the Elixhauser classification. | NR | 8796 | USA | Population-based | 5358 (61%) | NR | NR | NR |
| Turk et al.^50^ | Self-reported pain was defined as having pain in the head, neck, back, arm, hip, leg, or feet. | Self-report | 63 | USA | NR | 53 (84%) | Head 24%; back 22%; arm 19% | NR | NR |
| Van Der Slot et al.^29^ | Asked whether a person currently had pain. In participants with current pain, assessed per localization information on duration, frequency, and possible causes. Chronic pain defined as continuous or intermittent musculoskeletal or neuromuscular pain lasting longer than 3 months. | Self-report | 56 | Netherlands | Population-based | 75% | NR | NR | Pain lasting ≥1 year 68% |
| van Gorp et al.^1^ | Pain was assessed as the average pain severity over the previous week using an 11-point numerical rating scale ranging from 0 (no pain) to 10 (worst pain imaginable). Scores 1–3, mild; 4–7, moderate; 8–10, severe. | Self-report | 97 | Netherlands | Population-based | 28 (28%) (score >3) | NR | NR | NR |
| Veerbeek et al.^25^ | Self-developed questionnaire to indicate the frequency (‘never’, ‘occasionally’, ‘weekly’, and ‘daily’) and location (spinal level and upper and lower extremities) of pain experienced. | NR | 25 | South Africa | Clinic-based | NR | Low back 84% | Low back pain daily 28%; weekly 12%; occasionally 44%; never 16% | NR |
| Vidart et al.^6^ | Pain in previous week. | Self-report with or without assistance 68%; Proxy-report 32% | 164 | France, Germany, Sweden, Italy | Population-based | 117 (71%) | NR | Once or twice 22%; frequent 50% | NR |
| Whitney et al.^12^ | Identified the first claim for pain during the period of 1 October 2014 to 30 September 2015. Second, individuals had to have at least one more claim for pain on a subsequent day within 12 months after their first pain claim date in step 1, to rule out initial claims that may have been for screening. Third, categorized this sample on the basis of ‘new’ or ‘consistent’ pain using a look-back period of 12 months. New pain was defined as not having any claim for pain within 12 months before the first pain claim date in step 1; consistent pain was defined as having at least one claim for pain (any type or location) within 12 months before the first pain claim date in step 1. | NR | 5888 | USA | Population-based | 2162 (37%) | NR | NR | New pain 15%; consistent pain 22% |

^a^As a percentage of sample size unless stated otherwise.

^b^Reported as a percentage of total sample or mean/median in total sample unless stated otherwise.

^c^Among adults with pain.

^d^*n* = 96.

^e^*n* = 111.

Abbreviations: CI, confidence interval; NR, not reported; SF-36, 36-Item Short Form Survey; VAS, Visual Analog Scale.

**REFERENCES**

78. Benner JL, Hilberink SR, Veenis T, Stam HJ, van der Slot WM, Roebroeck ME. Long-Term Deterioration of Perceived Health and Functioning in Adults With Cerebral Palsy. Arch Phys Med Rehabil. 2017;98(11):2196-205.

79. Dauvergne F, Eon Y, Gallien P, Bouric S, Duruflé-Tapin A, Cambla N, Nicolas B. Disabilities, access to medical care, and way of life of adults with cerebral palsy. APIB study: first results. Ann Readapt Med Phys. 2007;50(1):20-27. doi:10.1016/j.annrmp.2006.06.008.

80. Botura C de A, Ames FQ, Botura AC de A, Bersani-Amado LE, Bardini AVSL, Cuman RK. Pain symptoms in patients with severe cerebral palsy: Prevalence among patients with higher degree of locomotor impairment. Trop J Pharm Res 2017;16(6):1431-6.

81. García Jalón EG, Maguire A, Perra O, Gavin A, O'Reilly D, Thurston A. Data linkage and pain medication in people with cerebral palsy: A cross-sectional study. Dev Med Child Neurol 2021;63(9):1085-92.

82. Marco Götze, MD, Andreas Geisbüsch, MD, Mirjam Thielen, MD, Leonhard Döderlein, MD, Sebastian I. Wolf, Thomas Dreher, MD, and Cornelia Putz, MD. Pain in Adults With Cerebral Palsy After Single-Event Multilevel Surgery.Am J Phys Med Rehabil 2022;101:119–123.

83. Sienko SE. An exploratory study investigating the multidimensional factors impacting the health and well-being of young adults with cerebral palsy. Disabil Rehabil 2018;40(6):660-6.

84. Thorpe D, Gannotti M, Peterson MD, Wang C-H, Freburger J. Musculoskeletal diagnoses, comorbidities, and physical and occupational therapy use among older adults with and without cerebral palsy. Disabil Health J 2021;14(2):101109.
