# Supplementary material for: Pain in adults with cerebral palsy: A systematic review
Source: Dev Med Child Neurol. 2025 Feb 12;67(7):854–74. doi: 10.1111/dmcn.16254 (PMC12134420; doi:10.1111/dmcn.16254)
Supplement: Supplementary file 13 — Table S10: Summary of clinical evidence profile for comparison: adults with cerebral palsy compared to adults without cerebral palsy. [file DMCN-67-854-s009.docx]

Supplemental table 10 Summary of clinical evidence profile for comparison: adults with cerebral palsy compared to adults without cerebral palsy

| Outcome | Illustrative comparative risk | Number of participants (studies) | Certainty in the evidence (GRADE) |
| --- | --- | --- | --- |
| Pain presence assessed using a variety of self-report questions or scales | Most studies found higher prevalence of pain among adults with CP.  Five studies showed greater prevalence of pain in adults with CP compared to adults without CP or reference values; one study found no difference. | 1,751 adults with CP^a^ (six observational studies) | Low  (due to methodological limitations and inconsistency) |

^a^number of adults without CP unknown
