# Supplementary material for: Pain in adults with cerebral palsy: A systematic review
Source: Dev Med Child Neurol. 2025 Feb 12;67(7):854–74. doi: 10.1111/dmcn.16254 (PMC12134420; doi:10.1111/dmcn.16254)
Supplement: Supplementary file 14 — Table S11: Description of prognostic factors for pain examined among adults with cerebral palsy. [file DMCN-67-854-s012.docx]

**Supplemental table 11. Description of prognostic factors for pain examined among adults with cerebral palsy**

| Author (year) | Pain assessment | n | Study design | Follow-up time^a^ | Socio-demographic factors description | CP-related factors description | Musculoskeletal factors description | Other factors description |
| --- | --- | --- | --- | --- | --- | --- | --- | --- |
| Boyer et al.^55^ | Hip pain; modified Harris Hip Score | 61 | cohort | Outcome assessed at 13-year follow-up |  |  |  | FDO between age 5 and 12 yr vs no FDO |
| Chin et al.^41^ | Pain intensity measured using PROMIS questionnaires | 17 | cross-sectional | Not applicable | Gender; household income; educational attainment | GMFCS; MACS; CFCS; ID |  | Catastrophising symptoms (Pain Catastrophising Scale);  anxiety/depression symptoms (Patient Health Questionnaire-4)  Number of orthopaedic surgeries  Current use of tone-altering medication |
| du Toit et al.^26^ | Frequency of pain was assessed based on a pain frequency questionnaire that asked participants to indicate how often they experience pain in their back (cervical, thoracic and lumbosacral regions), their upper limbs (shoulder and arm) and their lower limbs (leg, hip and knee). Responses were categorized as ‘never’, ‘occasionally’, ‘weekly’ and ‘daily’. The highest pain frequency was recorded for each level i.e., back, upper and lower limb. | 28 | cohort | Not reported |  |  |  | Satisfaction level with participation assessed using Assessment of Life Habits (Life-H) |
| Eken et al.^27^ | Frequency of pain was captured by asking participants to indicate how often they experienced pain in their back (head/neck, thoracic and lumbar spine level), upper limbs (shoulder, arm) and lower limbs (leg, hip, knee). The frequency was divided into ‘Never’, ‘Occasionally’, ‘Weekly’ and ‘Daily’. | 30 | cross-sectional | Not applicable |  |  |  | Accomplishment score on participation assessed using Assessment of Life Habits (Life-H) |
| Flanigan et al.^53^ | Brief Pain Inventory; pain in past week | 47 | cross-sectional | Not applicable |  | Self-ratings of spasms using the Penn Spasm Frequency Scale (PSFS) to rate the frequency of spasms on a 0 to 4 ordinal scale, with 0 indicating no spasms and 4 indicating spasms occurring more than 10 times per hour; Spasticity at elbow flexors, wrist flexors and knee extensors measured bilaterally using Modified Ashworth scale and summed |  |  |
| İçağasıoğlu et al.^43^ | Patient data were collected from the patients themselves and/or from their family members for those with speech disorder | 70 | cross-sectional | Not applicable | Age; education status (illiterate, literate, primary, secondary, high school or university graduate); accommodation status (living alone, with family, with spouse, with caregiver); employment status (unemployed, working part-time, working full-time) | ID (no, mild, moderate, severe) |  | comorbidity (presence or absence of epilepsy, speech disorder, sensory problems, respiratory problems, bladder/intenstinal problems, nutritional problems, skin problems) |
| Jacobson et al.^60^ | SF-36v2; "mild bodily pain" in past 4 weeks | 61 | cross-sectional | Not applicable | Sex |  | severe spasticity in lower limbs defined as Modified Ashworth Scale score ≥2 in one or several muscle groups | Fatigue assessed using Fatigue Severity Scale; sleep issues identified by asking participants to rate their general sleep quality and quantity as problematic or not, followed by questions on sleep medications |
| Jahnsen et al.^32^ | SF 36, bodily pain domain. Asked items on pain localisation, frequency and duration. Chronic pain defined as daily pain for 1 year or more. | 398 | cross-sectional | Not applicable | Age; sex; education (primary, secondary, university) |  |  | Physical role function (SF-36); life satisfaction (global item of the Life Satisfaction Scale); deterioration of skills (items on changes in locomotion skills/use of wheelchair during life); physical activity; fatigue (the Fatigue Questionnaire); emotional role function (SF-36) |
| Jarl et al.^36^ | NR | 408 | cross-sectional | Not applicable |  |  |  | HRQoL(EQ-5D-3L) |
| Jensen et al.^57^ | Current, worst, least, and average pain intensity (the latter three over the previous 3 months) were assessed using 11-point numerical rating scales, with 0 indicating “no pain” and 10 indicating “pain as bad as could be.” | 50 | cohort | Not reported |  |  |  | Time (0, 6, 12, 18, 24 months) |
| Jonsson et al.^34^ | Semi-structured interview; current or recurring pain of any type at the time of assessment Participants were asked to describe pain intensity, (mild, moderate, or severe), frequency (daily, weekly, monthly, or more seldom), and duration (more or less than 3 months). When participants reported several different pain sites, with different frequency, intensity, or duration, the highest intensity and frequency, and the longest duration was recorded. | 153 | cross-sectional | Not applicable |  | CP subtype; GMFCS level;  CFCS level; ID |  |  |
| Lundkvist and Westbom^56^ | Presence of pain was assessed by either the person him/herself or by proxy. For adults (> 18 years) recalling the last 4 weeks for spinal pain that was classified as being moderate or severe. | 66 | cohort | Outcome assessed at 20 years and 25 years |  |  |  | SDR at median age 4 yr (min 2.5 to max 6.6 yr) vs no SDR |
| Maanum et al.^52^ | Pain intensity was measured using the ‘bodily pain’ domain of the General Health Survey ShortForm 36 (SF-36), on a scale from 0 (worst pain) to 100 (no pain). | 126 | cross-sectional | Not applicable |  |  |  | 6MWT distance |
| Malone and Vogtle^49^ | Maximum pain intensity measured using the Wong-Baker FACES Pain Rating Scale. Pain locations documented using diagram of anterior and posterior human figures. | 26 | cohort | Not reported |  | Wheelchair use |  | Time (three assessments across three months) |
| Noonan et al.^54^ | Interview with primary caregiver; asked if, in his or her opinion, the hips of the subject were painful and frequency of pain. There were six potential answers: (1) the left hip was painful, (2) the right hip was painful, (3) both hips were painful, (4) at least one of the hips was painful but the caregiver was unsure which hip was painful, (5) the caregiver was not sure if the subject had any pain, and (6) neither hip was painful. | 77 | cross-sectional | Not applicable |  |  | Spasticity at knee graded from 0-4 on Ashworth scale; hip abduction; flexion contracture in at least one hip of <30°; windswept hips; hip dislocation and subluxation assessed using an anteroposterior pelvic radiograph |  |
| Opheim et al.^31^ | Self-reported questionnaire; the number of pain sites was reported with checkboxes for eight body parts, and pain frequency was reported as never, seldom, monthly, weekly, or daily. Daily pain for more than 1 year was considered chronic. | 149 | cohort | Outcome assessed at 7 year follow-up |  | Deterioration in walking function compared to improved/unchanged walking function over 7 years |  |  |
| Opheim et al.^44^ | Self-reported questionnaire; the number of pain sites was reported with checkboxes for eight body parts, and pain frequency was reported as never, seldom, monthly, weekly, or daily. Daily pain for more than 1 year was considered chronic. Pain intensity during the last month was assessed with a 100mm VAS with the anchor points ‘no pain’ and ‘worst possible pain’ | 149 | cross-sectional | Not applicable | Sex | Bilateral vs unilateral CP |  | Physical subscale of SF-36; mental subscale of SF-36 |
| Park and Kim^46^ | "general pain"; assessment not reported. | 52 | cross-sectional | Not applicable |  | GMFCS levels I-II vs III-V |  |  |
| Rodby-Bousquet et al.^42^ | Pain was reported either by client or by proxy as yes or no to any presence of pain during the last 4 weeks. | 102 | cross-sectional | Not applicable |  |  | Posture was assessed using items from the first version of the Posture and Postural Ability Scale called the Postural Ability Scale (PAS) |  |
| Rodby-Bousquet et al.^10^ | Prevalence of pain (Do you experience pain?) was either self- or proxy-reported as ‘yes’ or ‘no’. If pain was reported, pain severity was rated for the following 10 body sites: neck; back/spine; shoulder; arm/hand; hip/thigh; knee; feet/lower leg; head; stomach; or other location. As applicable, pain severity (How much bodily pain have you had during the past 4 weeks?) was graded according to the Short Form Health Survey 36 into one of the following response options for each relevant pain site: 1=none; 2=very mild; 3=mild; 4=moderate; 5=severe; 6=very severe. | 1591 | cross-sectional | Not applicable | Age; sex | GMFCS; CFCS; MACS; EDACS; CP-subtype |  |  |
| Sandstrom et al.^47^ | Pain localisation was described by means of given alternatives (neck, upper extremity, back, lower extremity). Pain intensity was rated on a visual analogue scale (VAS) from 0 mm (no pain) to 100 mm (worst imaginable pain). The individuals rated pain intensity as it was when it was at its worst and at its best. | 48 | cross-sectional | Not applicable |  | GMFCS | Range of motion in shoulder, elbow, wrist, hip, knee and ankle estimated using a scale from 0-2: 0=a few degrees, 1=limited ROM, 2=normal/almost normal ROM |  |
| Sienko^48^ | Magnitude of pain was assessed with the pain question from the 2011 Behavioral Risk Factor Surveillance System (BRFSS). The BRFSS pain question assesses pain on a five-point scale, with higher scores indicating more pain | 97 | cross-sectional | Not applicable |  | GMFCS |  |  |
| Terjesen et al.^51^ | "mild to moderate pain lasting several months in the back, hips or further distally"; assessment not reported. | 37 | cross-sectional | Not applicable |  | Mild disability (manage themselves and have good walking function); moderate/severe disability (need some help or help with almost everything and have reduced walking function or cannot walk) |  |  |
| Turk et al.^50^ | Self-reported Pain was defined as having pain in the head, neck, back, arm, hip, leg, or feet. | 63 | cross-sectional | Not applicable |  | ID |  |  |
| Van Der Slot et al.^29^ | Asked if a person currently had pain. In participants with current pain, assessed per localisation information on duration, frequency, and possible causes. Chronic pain defined as continuous or intermittent muscuoloskeletal or neuromuscular pain lasting longer than 3 months. | 56 | cross-sectional | Not applicable | Sex | GMFCS |  | Severity of fatigue (fatigue severity scale) |
| van Gorp et al.^40^ | Pain was assessed as the average pain severity over the past week using an 11-point numeric rating scale ranging from 0 (no pain) to 10 (worst pain imaginable). Scores 1-3 mild; 4-7 moderate; 8-10 severe. | 97 | cross-sectional | Not applicable |  |  |  | Fatigue; depressive symptoms; sleep disturbances (all assessed using PROMIS v1.0 short forms) |
| Yamashita et al.^45^ | Self-reported experience of any low back pain and, if present, record on a body chart where the pain was distributed. | 30 | cross-sectional | Not applicable |  | GMFCS; MACS | Postural ability in sitting; quality of sitting posture in frontal and sagittal view; all assessed using Posture and Postural Ability Scale | Self-reported body image of the low back (Fremantle Back Awareness Questionnaire; higher scores indicate more disturbed perception) |

^a^Follow-up time between prognostic factor and outcome assessment or timepoint of outcome assessment; CFCS: Communication Function Classification System; FDO: Femoral derotation osteotomy; GMFCS: Gross Motor Function Classification System; ID: Intellectual Disability; SD: standard deviation; SDR: selective dorsal rhizotomy; VAS: Visual Analog Scale; 6MWT: 6-minute walk test
