# Supplementary material for: Pain in adults with cerebral palsy: A systematic review
Source: Dev Med Child Neurol. 2025 Feb 12;67(7):854–74. doi: 10.1111/dmcn.16254 (PMC12134420; doi:10.1111/dmcn.16254)
Supplement: Supplementary file 15 — Table S12: 11 Summary of clinical evidence profile for comparison 2: GMFCS levels I–V. [file DMCN-67-854-s011.docx]

Supplemental table 12 Summary of clinical evidence profile for comparison 2: GMFCS levels I, II, III, IV and V

| Outcome | Illustrative comparative risk | Number of participants (studies) | Certainty in the evidence (GRADE) |
| --- | --- | --- | --- |
| Pain presence assessed using a variety of self-report questions or scales | Most studies found no difference in prevalence of pain across GMFCS levels.  Three studies found no difference in prevalence of pain across GMFCS levels. Two studies reported an association. | 1,882 (five observational studies) | Low  (due to methodological limitations and inconsistency) |
