# Supplementary material for: Pain in adults with cerebral palsy: A systematic review
Source: Dev Med Child Neurol. 2025 Feb 12;67(7):854–74. doi: 10.1111/dmcn.16254 (PMC12134420; doi:10.1111/dmcn.16254)
Supplement: Supplementary file 16 — Table S13: Summary of clinical evidence profile for comparison: CFCS levels I–V. [file DMCN-67-854-s018.docx]

Supplemental table 13 Summary of clinical evidence profile for comparison: CFCS levels I, II, III, IV and V

| Outcome | Illustrative comparative risk | Number of participants (studies) | Certainty in the evidence (GRADE) |
| --- | --- | --- | --- |
| Pain presence assessed using a variety of self-report questions | Prevalence of pain is higher among adults in lower CFCS levels | 1,744 (two observational studies) | Moderate  (due to methodological limitations) |
