# Supplementary material for: Pain in adults with cerebral palsy: A systematic review
Source: Dev Med Child Neurol. 2025 Feb 12;67(7):854–74. doi: 10.1111/dmcn.16254 (PMC12134420; doi:10.1111/dmcn.16254)
Supplement: Supplementary file 17 — Table S14: Summary of clinical evidence profile for comparison: pharmacological intervention compared to placebo. [file DMCN-67-854-s008.docx]

Supplemental table 14 Summary of clinical evidence profile for comparison: pharmacological intervention compared to placebo

| Outcome | Effect | Number of participants (studies) | Certainty in the evidence (GRADE) |
| --- | --- | --- | --- |
| Pain intensity assessed using numerical rating scale; short-term | No effect of Botulinum Toxin-A compared to placebo | 32 (two RCTs) | Low (due to methodological limitations and imprecision) |
| Pain intensity assessed using numerical rating scale; intermediate-term | No effect of Botulinum Toxin-A compared to placebo | 16 (one RCT) | Low (due to methodological limitations and imprecision) |
