# Supplementary material for: Pain in adults with cerebral palsy: A systematic review
Source: Dev Med Child Neurol. 2025 Feb 12;67(7):854–74. doi: 10.1111/dmcn.16254 (PMC12134420; doi:10.1111/dmcn.16254)
Supplement: Supplementary file 18 — Table S15: Summary of clinical evidence profile comparison: non‐pharmacological intervention compared to no intervention or usual care. [file DMCN-67-854-s002.docx]

Supplemental table 15 Summary of clinical evidence profile comparison: non-pharmacological intervention compared to no intervention or usual care

| Outcome | Effect | Number of participants (studies) | Certainty in the evidence (GRADE) |
| --- | --- | --- | --- |
| Pain assessed using SF-36; short-term | No effect of active lifestyle and sports participation intervention compared to usual care | 57 (one RCT) | Low (due to methodological limitations and imprecision) |
| Pain assessed using SF-36; long-term | Reduction in pain of 15.14 (95% CI 3.44 to 26.85) following active lifestyle and sports participation intervention compared to usual care | 57 (one RCT) | Low (due to methodological limitations and imprecision) |
| Pressure pain | Reduction in pain following somatosensory therapy in addition to standardised physical therapy for 12 weeks compared to standardised physical therapy (effect estimate not reported at 0 or 3 months post-intervention; “group × time × body interaction effect”, p < 0.05) | 32 (one RCT) | Low (due to methodological limitations and imprecision) |
