# Supplementary material for: Pain in adults with cerebral palsy: A systematic review
Source: Dev Med Child Neurol. 2025 Feb 12;67(7):854–74. doi: 10.1111/dmcn.16254 (PMC12134420; doi:10.1111/dmcn.16254)
Supplement: Supplementary file 19 — Table S16: Summary of clinical evidence profile comparison: surgical intervention compared to no intervention or usual care. [file DMCN-67-854-s001.docx]

Supplemental table 16 Summary of clinical evidence profile comparison: surgical intervention compared to no intervention or usual care

| Outcome | Effect | Number of participants (studies) | Certainty in the evidence (GRADE) |
| --- | --- | --- | --- |
| Pain intensity assessed using visual analogue scale; long-term | No effect of bilateral pallidal stimulation | 13 (one quasi-experimental study) | Very low (due to methodological limitations and imprecision) |
