# Supplementary figures and images for: Pain in adults with cerebral palsy: A systematic review
Source: Dev Med Child Neurol. 2025 Feb 12;67(7):854–74. doi: 10.1111/dmcn.16254 (PMC12134420; doi:10.1111/dmcn.16254)

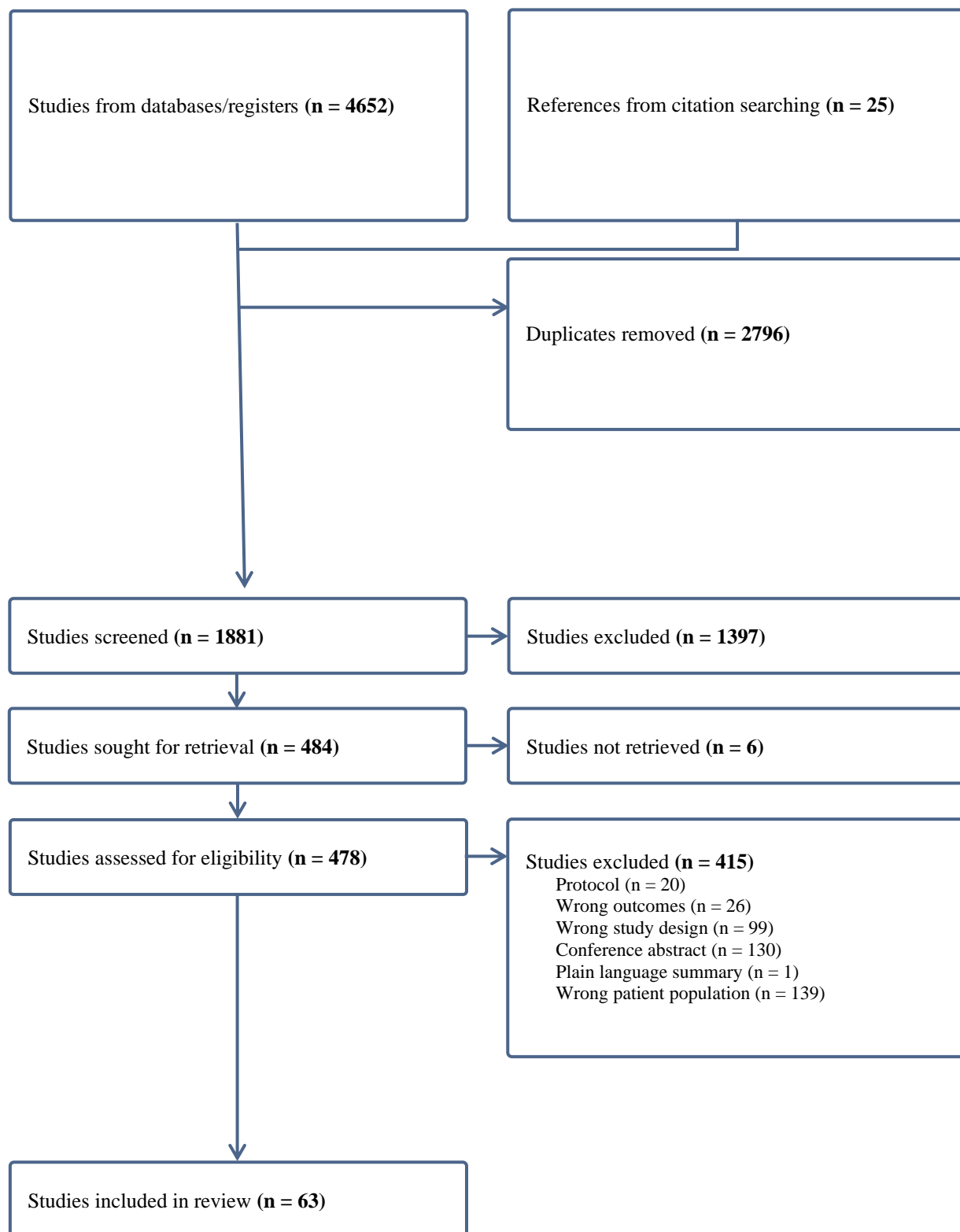

Figure S1 Flow diagram

Supplement: Supplementary file 20 — Figure S1: Flow diagram. [file DMCN-67-854-s007.pdf]
